# Achieving Image Encryption Quantum Dot‐Functionalized Encryption Camera with Designed Films

**DOI:** 10.1002/advs.202405667

**Published:** 2024-08-05

**Authors:** Xue Li, Tao Zhang, Mingriu Liu, Ying Fu, Haizheng Zhong

**Affiliations:** ^1^ School of Physics and Electronic Engineering Hebei Mizu Normal University Chengde 067000 China; ^2^ MIIT Key Laboratory for Low‐Dimensional Quantum Structure and Devices School of Materials Sciences & Engineering Beijing Institute of Technology Beijing 100081 China; ^3^ School of Computer Science and Technology Beijing Institute of Technology Beijing 100081 China; ^4^ School of Communication Engineering Hangzhou Dianzi University Hangzhou 310000 China

**Keywords:** camera, correlation, design QD films, image encryption, spatial redundancy

## Abstract

The risk of information leaks increases as images become a crucial medium for information sharing. There is a great need to further develop the versatility of image encryption technology to protect confidential and sensitive information. Herein, using high spatial redundancy (strong correlation of neighboring pixels) of the image and the in situ encryption function of a quantum dot functionalized encryption camera, in situ image encryption is achieved by designing quantum dot films (size, color, and full width at half maximum) to modify the correlation and reduce spatial redundancy of the captured image during encryption processing. The correlation coefficients of simulated encrypted image closely apporach to 0. High‐quality decrypted images are achieved with a PSNR of more than 35 dB by a convolutional neural network‐based algorithm that meets the resolution requirements of human visual perception. Compared with the traditional image encryption algorithms, chaotic image encryption algorithms and neural network‐based encryption algorithms described previously, it provides a universal, efficient and effective in situ image encryption method.

## Graphical abstract

The in situ image encryption is achieved through the design of quantum dots (QD) films, which involves reducing size, increasing color, and expanding full width at half maximum (FWHM) to modify the correlation between captured images and eliminate spatial redundancy. It provides a universal, efficient, and effective in situ image encryption method compared to other commonly symmetric encryption methods.

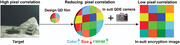

## 1 Introduction

The ubiquitous availability and rapid transmission of digital images have become an indispensable aspect of our daily lives in the digital era.^[^ ^1^, ^2^ ^]^ Whether they are personal photos or confidential business documents, protecting the confidentiality of these digital images has become a primary concern.^[^ ^3^ ^]^ Therefore, it is absolutely necessary to encrypt the image for security reasons. There are currently various image encryption technologies,^[^ ^1^ ^]^ such as traditional image encryption algorithms, chaotic image encryption algorithms and neural network‐based encryption algorithms. These techniques hardly meet the universal and efficient requirements of image capture. The original purpose of developing traditional image encryption (such as DEA,^[^ ^4^ ^]^ AES,^[^ ^5^ ^]^ DES,^[^ ^6^ ^]^ algorithm) was to secure text information.^[^ ^6^, ^7^ ^]^ However, image data inherently contains a significant amount of redundant information. The efficiency of traditional image encryption algorithm.^[^ ^4^, ^5^ ^]^ is limited due to inherent algorithmic limitation in image encryption. Although a chaotic image encryption algorithm is highly efficient in encrypting digital images, the use of high‐dimensional chaotic systems may increase the processing time and affect the practicality of encryption algorithm.^[^ ^7^, ^8^, ^9^, ^10^, ^11^ ^]^ Recent advances in artificial intelligence have led to the use of neural network‐based encryption algorithms in various image encryption tasks. The adaptability of neural networks enables dynamic adjustment of parameters based on image data characteristics and encryption requirements, increasing efficiency and versatility.^[^ ^12^, ^13^, ^14^ ^]^ However, limitations arise from factors such as network architecture and training data that affect the effective of these image encryption algorithms. Therefore, it is imperative to develop a universal, efficient and effectiveness image encryption method.

Digital images captured by the camera show distinct patterns in both the background and objects.^[^ ^15^, ^16^ ^]^ This indicates the strong correlation between neighboring pixels, which redundantly contributes to visual perception.^[^ ^15^, ^16^ ^]^ Therefore, the absence of this information does not affect human perception and understanding of the image content in the digital imaging process. Quantum dots (QDs) are color‐tunable fluorescent materials with sample patterning.^[^ ^17^, ^18^, ^19^, ^20^, ^21^, ^22^ ^]^ that enable spatial tuning of light intensity and colors of photoluminescence emission to modify correlation. According to the in‐situ encryption function of a quantum dot functionalized encryption camera (QDE camera),^[^ ^23^ ^]^ in situ encryption is achieved by integrating the fluorescence emission of QDs to scramble the spatial distribution of RGB values in the captured images during the imaging process. Herein, using high spatial redundancy (strong correlation of neighboring pixels) of the image and the in‐situ encryption function of QDE camera, we realize in situ image encryption by designing QD films (such as reducing the size, increasing the color, and broadening the full width at half maximum: FWHM). The in situ image encryption process changes the correlation and reduces the spatial redundancy of the captured image, achieving universal, efficient, and effective image encryption.

## 2 Result and Discussion

### 2.1 The Basic Principles of Encryption

**Figure 1:**
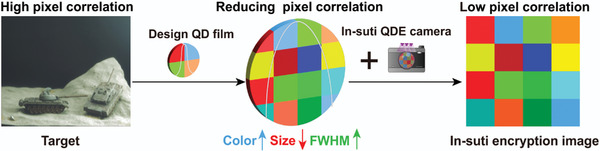
The schematic illustration of in situ image encryption based on QDE camera.

The QDE camera is constructed by integrating the QD films before the lens of the camera. The experimental setup and basic principles of the QDE camera have already been reported by our research group.^[^ ^23^ ^]^ This study investigates the influence of QD fluorescence on interpixel correlation in images captured with a QDE camera during the process of in situ image encryption. The basic principles of in situ image encryption using the QDE camera are shown schematically in **Figure** **1**. In a typical QDE camera system, the input information consists of the target to be encrypted and the QD film. The original image is captured by recording the intensity and color via a CCD sensor array (UV 356 nm: turn off). Under UV 365 nm light illumination, QDs can emit visible light with spatial distribution, which can be applied to scramble the light intensity and colors detected by the CCD. In situ encryption process is accomplished is achieved by coding the original image with additional calculation. Taking advantage of the strong correlation between neighboring pixels in the original image and the in situ encryption capability of the QDE camera, in situ encrypted image is obtained by reducing the size, increasing the colors and the FWHM of QD films (UV 356 nm: turn on). This is because these adjustments effectively reduce pixel correlation and eliminate spatially redundant information with image captured by the CCD sensor array. The supporting information provides a comprehensive description of the effects of QD film size, color, and FWHM during the in‐situ image encryption process.

### 2.2 The Design Analysis of Image Encryption

**Figure 2:**
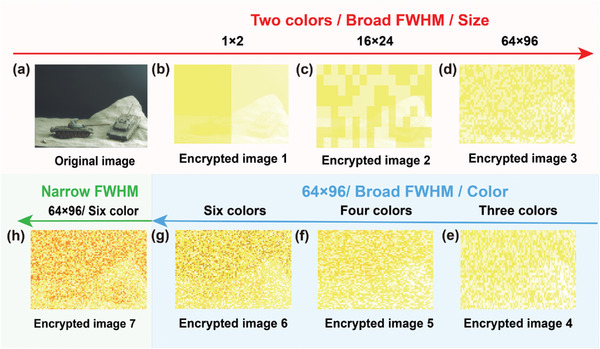
A design process for simulating in situ image encrypted based on QDE camera, the size of the image is 1392 × 1040 pixels and the pixel range of encrypted image is from 0 to 255. a) Desert scene is chosen as the original image. b–d) A series of simulated encrypted images with different sizes (broad FWHM/two colors/1 × 2, broad FWHM/two colors/16 × 24 and broad FWHM/two colors/64 × 96). e–g) A series of simulated encrypted images with different colors (64 × 96/broad FWHM/three colors, 64 × 96/broad FWHM/four colors and 64 × 96/broad FWHM/six colors). h) Simulated encrypted image using narrow FWHM (64 × 96/six colors).

The in situ image encryption effect of the QDE camera is affected by the size, color, and the FWHM of QD film. To verify the feasibility, an in situ image encryption design process is simulated to analyze how variations in these factors affect pixel correlation in captured images and image encryption results. The simulation results are shown in **Figure** **2**. The simulation of the in situ image encryption process is similar to the simulation of the QDE camera previously reported by our research group.^[^ ^23^ ^]^ A desert scene is selected as the original image, as shown in Figure 2a. The original image is divided into 1 × 2, 16 × 24, and 64 × 96 patches to study the influence of QD film size variation on pixel correlation in captured images and the in situ image encryption effect. Patches are randomly assigned two colors that match the original image, creating a series of key images (Figure S1, Supporting Information). The encrypted images are obtained by adding the original image (Figure 2a) to the corresponding key images using a saturated addition calculation, as shown in Figure 2b–d.

The encrypted images (Figure 2b–d) gradually lose distinguishable information as the patch size decreases. As shown in **Table** **1**, the encrypted image exhibits a reduction in correlations between neighboring pixels in the horizontal, vertical, and diagonal directions, respectively. Meanwhile, comparative analysis is performed using information entropy (Table S1, Supporting Information) and histograms (Figures S2 and S3, Supporting Information). The information entropy of the encrypted image exceeds that of the original image (Channel B, Table S1, Supporting Information). In addition, the information entropy of the encrypted image gradually increases as the patch size decreases. The results of the histogram analysis are consistent with the information entropy analyzes (Figures S2 and S3, Supporting Information). Similar results are obtained with increased color and FWHM of QD films, as shown in Figure 2d–h, Figures S4–S8 (Supporting Information), Table 1 and Tables S2–S4 Supporting Information). These phenomena indicate that universal, efficient and effective in situ image encryption can be achieved by adjusting the properties of QD film, such as reducing the size, increasing the color, and broadening the FWHM to minimize pixel correlation and spatial redundancy in the encrypted image.

**Table 1:** The correlation coefficients of the original image and encrypted image in the horizontal, vertical, and diagonal directions are measured for simulating in situ image encryption (QD films with different sizes, colors, and FWHM).

QD film (color/size/FWHM)	Original image			Encrypted image		
	Horizontal	Vertical	Diagonal	Horizontal	Vertical	Diagonal
Two /1 × 2/broad	0.9991	0.9875	0.987	0.9996	0.9994	0.9991
Two/16 × 24/broad	0.9991	0.9875	0.987	0.9996	0.9959	0.9956
Two /64 × 96/broad	0.9991	0.9875	0.987	0.9788	0.9746	0.9536
Three /64 × 96/broad	0.9991	0.9875	0.9870	0.9693	0.9624	0.8932
Four /64 × 96 /broad	0.9991	0.9875	0.9870	0.9622	0.9320	0.8923
Six /64 × 96/broad	0.9991	0.9875	0.9870	0.9603	0.9363	0.8914
Six /64 × 96/narrow	0.9981	0.9802	0.9791	0.9684	0.9319	0.8838

### 2.3 The Photoluminescence Properties of QD Films

**Figure 3:**
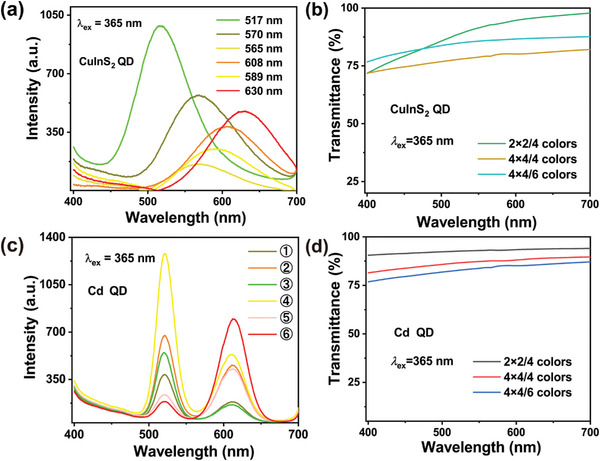
Photoluminescence emission spectra and transmittance spectra using different QD films. a,b) Photoluminescence emission spectra and transmittance spectra using different CuInS_2_ QD films. c,d) Photoluminescence emission spectra and transmittance spectra using different Cd QD films.

The reflectance spectrum of the desert scene covers all visible light (400–780 nm) with a central wavelength around 570 nm, as shown in Figure S9 (Supporting Information). The effectiveness of image encryption can be improved by using QD films that have a broad FWHM and color characteristics compatible with the desert scene (see Section S1 of the Supporting Information). The emission spectrum of CuInS_2_ QDs^[^ ^24^ ^]^ covers the entire visible light (500–780 nm) and exhibits characteristics such as broad FWHM (90–120 nm), large Stokes shift, and high fluorescence quantum yield (>80%). The photoluminescence emission spectrum of the CuInS_2_ QDs is essentially consistent with the reflection spectrum properties of desert scenes. Therefore, oil‐soluble CuInS_2_ QDs were selected as QD materials with broad FWHM, while Cd‐based QDs^[^ ^25^, ^26^ ^]^ are selected as QD materials with narrow FWHM for comparison in the experimental group. A series of QD films is prepared by a rationally designed spin coating method using PDMS.^[^ ^27^ ^]^ films as mask (see the Experimental Section). **Figure** **3** shows the photoluminescence emission spectra and transmittance spectra of fabricated QD samples. The resulting QD films have high transmittance spectra of over 75%, which only slightly affects the image acquisition of the target.

### 2.4 The Encryption Results

**Figure 4:**
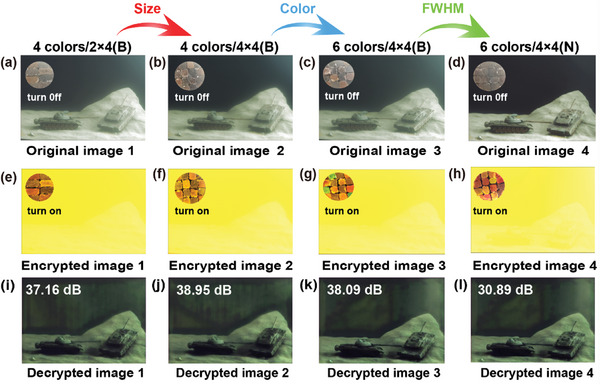
The encryption and decryption result of in situ image encryption with CuInS_2_ QD film. a–d) Original image (UV: turn off) with 4 colors/2 × 4 QD film, 4 colors/4 × 4 QD film and 6 colors/4 × 4 QD film and fabricated QD samples under daylight in the upper left corner. e–h) Encrypted image (UV: turn on) with 4 colors/2 × 4 QD film, 4 colors/4 × 4 QD film and 6 colors/4 × 4 QD film and fabricated QD samples under 365 nm UV lamp in the upper left corner. i‐l) The decryption results and PSNR of decryption image with CuInS_2_ QD films (4 colors/2 × 4 QD film, 4 colors/4 × 4 QD film and 6 colors/4 × 4 QD film), respectively.

**Figure** **4**a–h shows the original images (UV: turn off, Figure 4a–d) and encrypted images (UV: turn on, Figure 4e–h) that are obtained with four colors/2 × 4 CuInS_2_ QD film, four colors/4 × 4 CuInS_2_ QD film and six colors/4 × 4 CuInS_2_ QD film‐coated lens respectively. The upper left corner of each figure shows the photographs of QD film samples under daylight and a 365 nm UV lamp. The corresponding key image is shown in Figure S10 (Supporting Information), and the experimental results show that all the encrypted images (Figure 4e–h) do not reveal any identifiable information from the original image. However, the reduced size and increased color of the CuInS_2_ QD film led to a larger change in the pixel correlation between the original image and the encrypted image. This indicates a gradual decrease in correlation between neighboring pixels in the horizontal, vertical and diagonal directions, as shown in **Table** **2**. If high resolution patterned QD films can be developed with smaller size, more colors and broader FWHM, the correlation coefficients of encrypted image closely approximate 0, as shown in Figure S11 and Table S5 (Supporting Information). The information entropy of the original and encrypted images is calculated simultaneously (Table S6, Supporting Information). The difference in information entropy between the original image and the encrypted image gradually increases as the size of CuInS_2_ QD film decreases and its color increases. This observation suggests a reduction in pixel correlation and spatial redundancy in the encrypted image. Comparative analysis of histograms for encrypted images (Figure S12, Supporting Information) shows that the B channel gradually improves uniformity, which is consistent with the trends in information entropy. Similar results are obtained using narrow FHWM Cd‐based QD films as reference experiments (Figures S13–S15, Supporting Information).

**Table 2:** The correlation coefficients of the original image and encrypted image are measured along the horizontal (*H*), vertical (V), and diagonal (D) directions. (In situ image encryption; CuInS_2_ QD films with different sizes and colors).

QD film (color/size)	Original image			Encrypted image			Change in pixel correlation		
	*H*	*V*	*D*	*H*	*V*	*D*	ΔH	Δ*V*	Δ*D*
4/2 × 4	0.9985	0.9984	0.9978	0.9947	0.9979	0.9932	0.0038	0.0005	0.0046
4/4 × 4	0.9983	0.9990	0.9977	0.9945	0.9928	0.9907	0.0038	0.0062	0.0070
4/4 × 4	0.9974	0.9973	0.9974	0.9932	0.9907	0.9900	0.0042	0.0066	0.0074

A comparative analysis is conducted on the experimental results of CuInS_2_ and Cd QD films. The encrypted image obtained from CuInS_2_‐based QD films showed a greater change in pixel correlation (Table S7, Supporting Information) as well as information entropy (B channel, Figure S12, Supporting Information), compared to the encrypted image obtained from Cd‐based QD films (Table S8 and Figure S15, Supporting Information). The experimental results show that universal, efficient and effective in situ image encryption can be achieved by adjusting the properties of QD film, such as reducing the size, increasing the color, and broadening the FWHM, to minimize pixel correlation and spatial redundancy in the encrypted image. As shown in Figures S16–S25 (Supporting Information) and Tables S9–S16 (Supporting Information), similar encryption results can be achieved when other CuInS_2_ and Cd‐based QD films are used to encrypt other scenes.

### 2.5 The Decryption Results and Analysis

The encrypted image shows significant information loss in the G and R channels through entropy calculations and histogram analysis (Tables S6 and S8, Supporting Information; Figures S12 and S15, Supporting Information). Therefore, the convolutional neural network algorithm is used to obtain high quality decrypted images.^[^ ^28^ ^]^ The scene content of the original image can be accurately reconstructed by all decrypted images, as shown in Figure 4i–l. The intricate details are lost due to significant information loss in the R and G channels during the encryption process (Tables S6 and S8, Supporting Information; Figures S12 and S15, Supporting Information). However, recognition of scene content is not affected by the loss of image details when the human visual system acts as the image receiver. Meanwhile, this study also conducted quantitative analysis of the decrypted images by using the peak signal‐to‐noise ratio (PSNR).^[^ ^29^ ^]^ The PSNR value exceeded 35 dB using CuInS_2_ QD films as shown in Figure 4i–k. This indicates superior reconstruction quality of the decrypted images based on human visual perception. The PSNR value achieved with Cd QD films is 30.89 dB, as shown in Figure 4l. The use of Cd QD film leads to greater information loss in the encrypted image compared to CuInS_2_ QD film due to their FWHM influence on the encryption effect. Similar results are also obtained for other scenes such as desert scenes, forest scenes, grassland scenes, and woodland scenes (Figures S26–S29, Supporting Information and Table S17, Supporting Information).

## 3 Evaluation of Advantages and Performance

In situ image encryption is a type of symmetric encryption. Therefore, its advantages (such as universality, effectiveness and efficiency) are compared with other commonly symmetric encryption methods, as shown in **Table** **3**. Each image includes different types (such as text and RGB images) and formats (JPEG, PNG) with distinctive features. It remains a complex challenge to develop an image encryption technology that support different types and formats while maintaining its security properties. The in situ image encryption has superior universality and encryption effectiveness compared to other symmetric algorithms, allowing image encryption to be performed efficiently (0.032 s).

**Table 3:** The comparison between in situ image encryption with DES/AES/S‐box, chaos‐based encryption algorithm, and neural network‐based encryption algorithm.

Method	Type	Format	Adaptability	Efficiency	Effectiveness
DES/AES/S‐Box^[^ ^1^, ^5^, ^6^ ^]^	Text	ASCII	No	Good	Yes
Chaos^[^ ^1^, ^7^, ^8^, ^9^, ^10^ ^]^	Text/image	JPEG/PNG	Yes	Good (1/2D)	Restricted
Neural network^[^ ^1^, ^12^, ^13^, ^14^ ^]^	Text/image	JPEG/PNG	Yes	Good	Restricted
Our work	Text/image	JPEG/PNG	Yes	Good	Yes

According to the basic principles of QDE camera,^[^ ^23^ ^]^ image encryption can be achieved when the information entropy of the key image exceeds that of the original image during the encryption process.^[^ ^15^ ^]^ Therefore, the performance of in‐situ image encryption is evaluated using information entropy (Table S18, Supporting Information).^[^ ^29^, ^30^, ^31^ ^]^ The information entropy of the original image and the key image is calculated. The results show that the key image has lower information entropy compared to the original image. However, it is difficult for humans to distinguish the content of the original image from encrypted images (Figure 4e–h) based on visual perception. This can be attributed to information loss during encryption process with QDE cameras, which hides pixel correlations of encrypted images (Figure 4e–h, Figure S14d–f, Supporting Information and Tables S6 and S8, Supporting Information). Image encryption is done by leveraging visual masking effects on human vision. Due to the increased information entropy of one channel in encrypted images (B channel, Tables S6 and S8, Supporting Information), high quality decrypted images based on convolutional neural network‐based algorithms can still be obtained. Similar results are observed in different scenarios such as desert, forest, grassland, and woodland scenes, as shown in Tables S18–S22 (Supporting Information). These results show that it is possible to achieve universal, efficient, and effective in‐situ image encryption visually by designing films based on QDE camera.

## 4 Conclusion

In summary, it exploits the high spatial redundancy of the image (strong correlation between neighboring pixels) and the in situ encryption properties of the QDE camera. In situ image encryption is achieved visually through the design of QD films, such as reducing the size, increasing the color and expanding the FWHM. This alteration effectively modifies the correlation between captured images and eliminates spatial redundancy. Through using CuInS_2_ QD films, high quality decrypted images are achieved with a PSNR greater than 35 dB, which meets the resolution requirements of human visual perception. A universal, efficient, and effective in‐situ image encryption technology is achieved compared to other commonly symmetric encryption methods. It provides a promising cloud encryption technique to protect information confidentiality and security during the development of digitization.

## 5 Experimental Section

### The Preparation of QD Films

CuInS_2_ QD, CdZnSeS/ZnS QD, and CdSe/CdS/ZnS QD were prepared in the lab by adapting a reported procedure.^[^ ^24^, ^25^, ^26^ ^]^ 1 g PDMS (A:B = 1:10) was mixed with a fixed amount of QD solution to form a precursor solution, which was placed for 1–2 h for defoaming. To prepare QD film with different size, color, and FWHM, a preformed PDMS was used as a template to protect the fixed region of the glass lens. QD film was fabricated by spin coating the QD precursor solutions on the unprotected region using different color and FWHM subsequently. After that, the as‐fabricated QD film was heated at 100 °C for 3–5 min for further use.

### QD Film Characterization

The photoluminescence spectra were obtained using a fluorescence spectrometer (F‐380, Tianjin Gangdong Technology, range:200‐1000). UV–vis diffuse reflection spectra were measured using a spectrophotometer (UV‐6100, Shanghai Mapada Instruments Co., Ltd., range: 300–1100 nm).

### Digital Image Acquisition

The key images and encrypted images were recorded using a camera (CM‐140GE, *f* = 35 mm) with QD film coated lens under the illumination of a UV 365 nm LED lamp. The original image was collected when the UV LED lamp was turned off.

### Decryption Process: Network

To obtain high‐quality decryption image, a deep decryption network was employed.^[^ ^29^ ^]^ The input of the network is the concatenation of ciphertext and key images, and the output is plaintext image. The overall structure is based on typical Unet architecture, which consists of 4 encoder stages and 4 corresponding decoder stages. At the end of each encoder stage, the feature maps are downsampled to 1/2 scale with a 4 × 4 kernel size and 2 stride convolution. Before each decoder stage, the feature maps are upsampled to 2 scale with bilinear interpolation. Skip connections pass large‐scale low‐level feature maps from each encoder stage to its corresponding decoder stage. To ease the training, residual learning is introduced into the network. Specifically, residual block is utilized as the fundamental block to build encoder and decoder. The residual block is conducted by two 3 × 3 convolutions followed by ReLU activation function and a 1 × 1 convolution.

### Dataset

To training the decryption network, a dataset with paired plaintext, key, and ciphertext images is collected.

### Training

In the training stage, overlapped 256 × 256 spatial regions are randomly cropped from images in the paired dataset. This implementation is based on PyTorch. The models are trained with Adam optimizer (*β*_1_ = 0.9 and *β*_2_ = 0.999) for 100 epochs. The initial learning rate and mini‐batch size is set to 1 × 10^‐4^ and 1, respectively.

## Conflict of Interest

The authors declare no conflict of interest.

## Author Contributions

H.Z. and Y.F. conceived and supervised the project. X.L. fabricated the patterned QD films, conducted the QD film characterizations, and recorded the digital images. T.Z. and Y.F. performed the algorithm derivation. M.L. fabricated CuInS_2_ QD, CdZnSeS/ZnS QD and CdSe/CdS/ZnS QD. X.L. and H.Z. analyzed data. H.Z., Y.F., and X.L. wrote the paper, which was then discussed with T.Z.

## Supporting information

Supporting Information

## Data Availability

The data that support the findings of this study are available in the supplementary material of this article.

## References

[1] M. SaberiKamarposhti , A. Ghorbani , M. Yadollahi , Chaos, Solitons Fractals 2024, 178, 114361.

[2] Priyanka , N. Baranwal , K. N. Singh , A. K. Singh , Future Gener. Comput. Syst. 2024, 150, 1.

[3] M. Qin , Q. Lai , Appl. Math. Modell. 2024, 125, 125.

[4] D. Kahn , The Codebreakers: The Comprehensive History of Secret Communication from Ancient Times to the Internet, Simon and Schuster, New York 1996.

[5] A. M. Abdullah , Cryptography and Network Security 2017, 16, 1.

[6] J. O. Grabbe , Laissez Faire City Times 2010, 28, 1.

[7] Y. Wu , X. Dai , J. Intell. Fuzzy Syst. 2020, 39, 5085.

[8] E. Gokcay , H. Tora , Expert Syst. Appl. 2024, 237, 121494.

[9] I. Ahmad , Appl. Math. Comput. 2021, 395, 125858.

[10] Y. Khedmati , R. Parvaz , Y. Behroo , Inf. Sci. 2020, 512, 855.

[11] A. A. Rezk , A. H. Madian , A. G. Radwan , A. M. Soliman , AEU‐Int. J. Electron. Commun. 2020, 113, 152947.

[12] X. Y. Wang , S. Gao , Inf. Sci. 2020, 507, 16.

[13] J. Xie , K. Hu , G. Li , Y. Guo , Expert Syst. Appl. 2021, 169, 114442.

[14] Y. Zhang , A. Chen , Y. Tang , J. Dang , G. Wang , Inf. Sci. 2020, 526, 180.

[15] C. E. Shannon , Bell Syst. Tech. J. 1949, 28, 656.

[16] R. C. Gonzalez , R. E. Woods , Digital Image Processing, 4th ed., Pearson, New York, NY 2018.

[17] Y. Liu , F. Han , F. Li , Nat. Commun. 2019, 10, 2409. 31160579 10.1038/s41467-019-10406-7 PMC6547729

[18] W. Huang , M. Xu , J. Liu , Adv. Funct. Mater. 2019, 29, 1808762.

[19] C. Sun , S. Su , Z. Gao , ACS Appl. Mater. Interfaces 2019, 11, 8210. 30719905 10.1021/acsami.8b19317

[20] J. Wang , C. F. Wang , S. Chen , Angew. Chem., Int. Ed. Eng. 2012, 51, 9297. 10.1002/anie.201204381 22907831

[21] A. P. Alivisatos , Science 1996, 271, 933.

[22] M. V. Kovalenko , L. Manna , A. Cabot , ACS Nano 2015, 91012.

[23] X. Li , J. Peng , T. Zhang , M. R. Liu , Y. Fu , H. Z. Zhong , J. Zhang , Adv. Intell. Syst. 2022, 4, 202200171.

[24] H. Z. Zhong , M. F. Ye , Y. J. He , J. P. Ye , Y. F. Li , Chem. Mater. 2008, 20, 6434.

[25] K. H. Lee , J. H. Lee , H. D. Kang , ACS Nano 2014, 8, 4893. 24758609 10.1021/nn500852g

[26] K. Kumagai , T. Uematsu , T. Torimoto , Chem. Mater 2020, 33, 1607.

[27] F. Schneider , J. Draheim , R. Kamberger , Sens. Actuators, A 2009, 151, 95.

[28] O. Ronneberger , P. Fischer , T. Brox , in Navab N. , Hornegger J. , Wells W. , Frangi A. (eds) Proceedings of the Medical Image Computing and Computer‐Assisted Intervention‐MICCAI 2015: 18th Int. Conf., Springer, Munich 2015, pp. 234–241.

[29] M. Kaur , V. Kumar , Arch. Comput. Methods Eng. 2018, 27, 15.

[30] N. Chidambaram , K. Thenmozhi , P. Raj , R. Amirthatajan , Cluster Comput 2024, 10.1007/s10586-024-04391-w.

[31] S. A. Banu , A. I. Al‐Alawi , M. Padmaa , P. S. Priya , V. Thanikaiselvan , R. Amirtharajan , Multimedia Tools Appl. 2024, 83, 21153.

