# Supplementary material for: Achieving Image Encryption Quantum Dot‐Functionalized Encryption Camera with Designed Films
Source: Adv Sci (Weinh). 2024 Aug 5;11(38):2405667. doi: 10.1002/advs.202405667 (PMC11481269; doi:10.1002/advs.202405667)
Supplement: Supplementary file 1 — Supporting Information [file ADVS-11-2405667-s001.pdf]

ADVANCED SCIENCE Open Access

Supporting Information

for *Adv. Sci.*, DOI 10.1002/advs.202405667

Achieving Image Encryption Quantum Dot-Functionalized Encryption Camera with Designed Films

Xue Li, Tao Zhang, Mingriu Liu, Ying Fu and Haizheng Zhong**

Achieving Image encryption Quantum Dot-Functionalized Encryption Camera with Designed films

Xue Li[1,2], *Tao Zhang*[3,4], *Mingrui Liu*[2], *Ying Fu*[3*], *Haizheng Zhong*[2*]

[1]School of Physics and Electronic Engineering, Hebei MinZu Normal University, Chengde 067000, China

[2]MIIT Key Laboratory for Low-dimensional Quantum Structure and Devices, School of Materials Sciences & Engineering, Beijing Institute of Technology, 100081 Beijing, China.

[3]School of Computer Science and Technology, Beijing Institute of Technology, 100081 Beijing, China.

[4]School of Communication Engineering, Hangzhou Dianzi University, 310000 hangzhou, China.

Correspondence:

Haizheng Zhong, *E-mail: hzzhong@bit.edu.cn Ying Fu, *E-mail: fuying@bit.edu.cn

1. The theoretical foundations of in situ image encryption.

The definition of redundancy can described be using Equation (1)

$$\gamma = 1 - \frac{H_\infty}{H_0} \tag{1}$$

Where, H_∞ represents the extreme entropy, which is the actual information entropy of the source. H_0 represents the average information entropy. γ represents redundancy.

The definition of redundancy (Equation 1) suggests that a digital image contains both essential and redundant data. Spatial redundancy primarily arises from the correlation between neighboring pixels in images, where these neighboring pixels often have similarities or slight difference in values. Consequently, many pixels redundantly contribute to visual perception because their values can be derived from neighboring pixels. The image dataset is primarily composed of various terrain images, including Gobi, forest, grassland, and woodland terrains in this study. The collected images have slight variations in pixel values between neighboring pixels. This indicates the presence of significant spatial redundancy. Image encryption technology uses permutation or substitution operations to hide or eliminate the correlation between neighboring pixels in the original image, thereby reducing spatial redundancy and achieving encryption of the original image.

In the QDE camera, the fluorescence information from QDs is used to replace the original image pixel values. This process effectively hides or eliminates the correlation in the original image while also removing its spatial redundancy. Consequently, the information entropy of original image it significantly improved to achieve image encryption. The loss of spatially redundant information does not have a significantly impact on human recognition of image content. Therefore, it becomes possible to manipulate QD fluorescence information to hide or eliminate the correlation between neighboring pixels in images by utilizing the high spatial redundancy properties of the image and the in situ encryption capabilities of the QDE camera. This allows for hiding or eliminating spatially redundant information in an image, thereby facilitating in situ image encryption.

2. Factors affecting the effectiveness of image encryption

Spatial redundancy arises from the strong correlation between neighboring pixels in an image based on the theoretical foundations of in situ image encryption. The correlation between neighboring pixels can be affected by factors such as size,

color, and FWHM of the QD film in an image. This reduces the effectiveness of in-situ encryption images.

The influence of QD film size. The original image is divided into P×P squares using QD film during the QDE camera's encryption process. The QD film's fluorescence is used to equally replace all pixel values within each square, disrupting the pixel correlation in the original image. This hides or eliminates the spatial redundancy of the image, resulting in an increase in information entropy. This enables in-situ image encryption. The correlation between pixels is affected by the size of the QD film in an image based on the encryption principle of the QDE camera. The correlation between neighboring pixels is maintained within a square as long as the sum of the pixel values does not exceed 255 (8-bit image). Because the difference between neighboring pixels remains unchanged. However, if certain pixels within the square exceed the value of 255, the pixel correlation is weakened by changing the difference between neighboring pixels. The pixel information is completely changed to zero, resulting in a reduction of the information entropy within the square to zero when all pixel values are 255 (8-bit). Therefore, the preservation of pixel correlation in the image improves proportionally with the enlargement size of the QD film.

The influence of QD film color. The in situ image encryption effect is influenced by the color of the QD film. Equation 2 can be used to record the pixel value at a given pixel point based on the QDE camera.

$$r(x,y)=\int_{\lambda_{\min}}^{\lambda_{\max}}(D_{\lambda}(x,y)+QD_{\lambda}(x,y))S_{\lambda}(x,y)d_{\lambda} \qquad (2)$$

Where, $r(x,y)$ represents the pixel value of the captured image at a given pixel point. $QD_{\lambda}(x,y)$ represents the pixel value of QD film at a given pixel point. $S_{\lambda}(x,y)$ represents the spectrum of the target at a given point, while $D_{\lambda}(x,y)$ represents the response curve of the camera. The pixel range can be adjusted by increasing the color of QD film within a given square, thereby reducing the correlation between neighboring squares and minimizing spatial redundancy to increase the effectiveness of image encryption.

Furthermore, it is observed that the original image (topographic image) displays a consistent color tone with minimal variations between adjacent pixels. When the color of the QD film is similar to that of the original image content (topographic image), it only requires a slight perturbation in the fluorescence intensity of QDs to

make the pixel values of the original image identical or very close. Due to the brightness masking effect of human eyes, it becomes difficult for the visual system to distinguish pixel values differences in the image. As a result, encryption effect of images is improved visually.

The human visual system consists of three types of cone cells, namely red (R), green (G), and blue (B). These cones have different levels of sensitivity. The sensitivity to red and green light is 20 times higher compared to that of blue light. The human brain perceives color tones and saturation based on their relative proportions, while the overall brightness is determined by the combined values. The spectral response curves of visible CCD cameras are carefully designed to closely mimic the human eye's perception. Therefore, it becomes difficult to achieve a visual encryption effect utilizing brightness masking for images with a higher proportion of blue light.

The influence of QD material's FWHM. According to formula 2, wider FWHM of QD materials leads to significant pixel value variation within a given square compared to narrower FWHM QD materials. This effectively reduces the correlation between adjacent squares, thereby eliminating spatial redundancy in the image and enhancing its encryption effectiveness.

Table S1. Information entropy of original image and encrypted image using QD film of different sizes (simulating in situ image encryption)

Size	Original image H（2D）			Encrypted image H（2D）		
	R	G	B	R	G	B
Broad FWHM/Two colors/1×2	7.2434	7.8571	6.9991	0	0	7.2548
Broad FWHM/Two colors/16×24				0	0	7.7212
Broad FWHM/Two colors/64×96				0	0	8.0407

Table S2. Information entropy of original image and encrypted image using QD film of different colors (simulating in situ image encryption).

Color	Original image H（2D）			Encrypted image H（2D）		
	R	G	B	R	G	B
Broad FWHM/Three colors/64×96	7.2434	7.8571	6.9991	0	0	9.0336
Broad FWHM/Four colors/64×96				0	1.9148	9.4034
Broad FWHM/Six colors/64×96				1.3912	4.1504	9.6585

Table S3. Alteration in pixel correlation between original image and encrypted image using QD film of different FWHM (Six colors/64×96, simulating in situ image encryption).

FWHM	Alteration in pixel correlation		
	Horizontal	Vertical	Diagonal
Narrow	0.0297	0.0318	0.0953
Broad	0.0388	0.0512	0.0956

Table S4. Information entropy of original images and encrypted images using QD film of different FWHM (Six colors/64×96, simulating in situ image encryption).

FWHM	Original image H（2D）			Encrypted image H（2D）			ΔH（2D）		
	R	G	B	R	G	B	R	G	B
Narrow	6.5880	7.1946	6.5063	0	5.2729	9.0873	-6.5880	-1.9217	2.5810
Broad	7.2434	7.8571	6.9991	1.3912	4.1504	9.6585	-5.8522	-3.7067	2.6594

Table S5. The correlation coefficients of the original image and encrypted image in the horizontal, vertical, and diagonal directions using high resolution patterned QD film.

Image	correlation coefficients		
	Horizontal	Vertical	Diagonal
Original image	0.9991	0.9875	0.987
Encrypted image	0.0125	0.0157	0.0152

Table S6. Information entropy of original images and encrypted images using $CuInS_2$ QD films of different sizes and colors (in situ image encryption)

Film	Original image H（2D）			Encrypted image H（2D）			ΔH（2D）		
	R	G	B	R	G	B	R	G	B
4colors/2×4	7.4015	8.0455	7.2109	0	0.0182	7.5564	-7.4015	-8.0273	0.0155
4colors/4×4	7.2073	7.8450	7.0290	0	0.0718	7.5654	-7.2073	-7.7732	0.5364
6colors/4×4	7.0900	7.7008	6.8931	0	0.1244	7.8523	-7.0900	-7.5764	0.9592

Table S7. The correlation coefficients of the original image and encrypted image are measured along the horizontal (H), vertical (V), and diagonal (D) directions. (in situ image encryption; Cd QD films with different sizes and colors).

Film	Original image			Encrypted image			Change in pixel correlation		
	H	V	D	H	V	D	ΔH	ΔV	ΔD
4colors/2×4	0.9956	0.9982	0.9943	0.9985	0.9981	0.9910	-0.0029	0.0001	0.0033
4colors/4×4	0.9990	0.9986	0.9982	0.9956	0.9985	0.9948	0.0034	0.0001	0.0034
4colors/4×4	0.9980	0.9981	0.9966	0.9939	0.9979	0.9922	0.0041	0.0002	0.0044

Table S8. Information entropy of original images and encrypted images (in situ image encryption; Cd QD films with different sizes and colors).

Film	Original image H（2D）			Encrypted image H（2D）			ΔH（2D）		
	R	G	B	R	G	B	R	G	B
4colors/2×4	7.3551	7.7901	7.2051	0	4.4419	7.2204	-7.3551	3.3482	0.0153
4colors/4×4	7.3470	7.8874	7.2351	0	0.0020	7.6634	-7.3470	-7.8854	0.2240
6colors/4×4	7.0343	7.4484	7.1261	0	1.1205	8.0102	-7.0343	-6.3279	0.8841

Table S9. The correlation coefficients of the original image and encrypted image are measured along the horizontal (H), vertical (H), and diagonal (H) directions (Gobi scene; in-situ image encryption; $CuInS_2$ and Cd QD films with different sizes and colors).

QD	Film	Original image			Encrypted image		Change in pixel correlation			
		H	V	D	H	V	D	ΔH	ΔV	ΔD
$CuInS_2$	4colors/2×4	0.9958	0.9943	0.9922	0.9940	0.9933	0.9894	0.0018	0.0010	0.0028
	4colors/4×4	0.9984	0.9952	0.9947	0.9911	0.9924	0.9841	0.0073	0.0028	0.0106
	4colors/4×4	0.9924	0.9955	0.9887	0.9654	0.9807	0.9487	0.0270	0.0148	0.0400
Cd	4colors/2×4	0.9973	0.9964	0.9952	0.9968	0.9958	0.9940	0.0005	0.0006	0.0012
	4colors/4×4	0.9982	0.9972	0.9966	0.9960	0.9966	0.9938	0.0022	0.0006	0.0028
	4colors/4×4	0.9966	0.9952	0.994	0.9828	0.9945	0.9789	0.0138	0.0007	0.0151

Table S10. Information entropy of original images and encrypted images using $CuInS_2$ and Cd QD films with different sizes and colors (Gobi scenes, in-situ natural image encryption).

QD	Film	Original image H（2D）			Encrypted image（2D）			ΔH（2D）		
		R	G	B	R	R	G	B	G	R
$CuInS_2$	4colors/2×4	6.0733	6.5765	5.9971	0	0.0434	7.2560	-6.0733	-6.5531	1.2589
	4colors/4×4	6.1581	6.6876	6.0362	0	0	7.3492	-6.1581	-6.6876	1.3130
	6colors/4×4	5.9540	6.4943	5.6814	0	0	7.4036	-5.9540	-6.4943	1.7222
Cd	4colors/2×4	5.8551	6.1740	5.6961	0	3.8528	6.6646	-5.8551	-2.3212	0.9685
	6colors/4×4	5.8015	6.1482	6.0449	0	0	7.3348	-5.8015	6.1482	1.2902
	6colors/4×4	6.0381	6.4129	5.9093	0	0.0215	7.3459	-6.0381	-6.3914	1.4352

Table S11. The correlation coefficients of the original image and encrypted image are measured along the horizontal (H), vertical (H), and diagonal (H) directions (Forest scenes; in-situ image encryption; $CuInS_2$ and Cd QD films with different sizes and colors).

QD	Film	Original image			Encrypted image			Change in pixel correlation		
		H	V	D	H	V	D	ΔH	ΔV	ΔD
$CuInS_2$	2colors/1×2	0.9895	0.9888	0.9811	0.9833	0.9748	0.9701	0.0062	0.0140	0.0101
	3colors/1×3	0.9953	0.9947	0.9927	0.9757	0.9943	0.9738	0.0196	0.0010	0.0189
	4colors/2×2	0.9947	0.9889	0.9855	0.9072	0.7966	0.6850	0.0875	0.1923	0.3005
Cd	2colors/1×2	0.9971	0.9833	0.9807	0.9938	0.9825	0.9742	0.0033	0.0008	0.0091
	3colors/1×3	0.9960	0.9843	0.9819	0.9922	0.9684	0.9636	0.0038	0.0159	0.0183
	4colors/2×2	0.9940	0.9945	0.9901	0.9840	0.9498	0.9455	0.0100	0.0477	0.0446

Table S12. Information entropy of original images and encrypted images using $CuInS_2$ and Cd QD films with different sizes and colors (Forest scenes, in-situ image encryption).

QD	Film	Original image H（2D）			Encrypted image （2D）			ΔH（2D）		
		R	G	B	R	R	G	B	G	R
$CuInS_2$	2colors/1×2	6.3717	7.5656	6.1382	1.971	0	8.3741	-4.4007	-7.5656	2.2359
	3colors/1×3	6.3617	7.5519	6.2083	4.7044	0	8.4843	-1.6573	-7.5519	2.2760
	4colors/2×2	6.2771	7.4460	5.8000	5.5026	0	8.0788	-0.7745	-7.4460	2.2788
Cd	2colors/1×2	6.7412	7.8776	6.8751	6.4527	0	8.1950	-0.2885	-7.8776	1.3199
	3colors/1×3	6.4761	7.6628	6.5957	6.0248	0	8.6569	-0.4513	-7.6628	2.0612
	4colors/2×2	6.5332	7.6518	6.4777	6.3368	0	8.6140	-0.1964	-7.6518	2.2772

Table S13. The correlation coefficients of the original image and encrypted image are measured along the horizontal (H), vertical (H), and diagonal (H) directions (Grassland scenes; in-situ image encryption; $CuInS_2$ and Cd QD films with different sizes and colors).

QD	Film	Original image			Encrypted image			Change in pixel correlation		
		H	V	D	H	V	D	ΔH	ΔV	ΔD
$CuInS_2$	2colors/1×2	0.9875	0.9909	0.9799	0.9969	0.9541	0.9520	-0.0094	0.0368	0.0279
	3colors/1×3	0.9905	0.9960	0.9886	0.8959	0.9533	0.7927	0.0946	0.0042	0.1959
	4colors/2×2	0.9958	0.9912	0.9896	0.9103	0.9853	0.7065	0.0855	0.0057	0.2831
Cd	2colors/1×2	0.9963	0.9822	0.9800	0.9997	0.9522	0.9632	-0.0034	0.0300	0.0168
	3colors/1×3	0.9822	0.9866	0.9702	0.9961	0.9773	0.9944	-0.0139	0.0159	0.0242
	4colors/2×2	0.9952	0.9890	0.9803	0.9743	0.9833	0.8652	0.0209	0.0057	0.1151

Table S14. Information entropy of original images and encrypted images using $CuInS_2$ and Cd QD films with different sizes and colors (Grassland scenes, in-situ image encryption).

QD	Film	Original image H（2D）			Encrypted image （2D）			ΔH（2D）		
		R	G	B	R	R	G	B	G	R
$CuInS_2$	2colors/1×2	5.8890	6.007	5.6380	2.9673	0.0196	7.0729	-2.9217	-5.9874	1.4349
	3colors/1×3	5.9946	6.7181	5.7760	0.0190	0	7.6565	-5.9756	-6.7181	1.8805
	4colors/2×2	5.7477	6.4299	5.5131	3.5716	0	7.4990	-2.1761	-6.4299	1.9859
Cd	2colors/1×2	6.8399	7.7849	6.7350	3.4329	0	7.3341	-3.4070	-7.7849	0.5991
	3colors/1×3	6.7496	7.6099	6.5058	1.3111	0	7.8703	-5.4385	-7.6099	1.3645
	4colors/2×2	6.6271	7.6112	6.5660	2.1881	0	7.9838	-4.4390	-7.6112	1.4178

Table S15. The correlation coefficients of the original image and encrypted image are measured along the horizontal (H), vertical (H), and diagonal (H) directions (Woodland scenes; in-situ image encryption; $CuInS_2$ and Cd QD films with different sizes and colors).

QD	Film	Original image			Encrypted image			Change in pixel correlation		
		H	V	D	H	V	D	ΔH	ΔV	ΔD
$CuInS_2$	2colors/1×2	0.9974	0.9951	0.9941	0.9871	0.9879	0.9755	0.0103	0.0072	0.0186
	3colors/1×3	0.9963	0.9896	0.9867	0.9845	0.9961	0.9723	0.0118	-0.0065	-0.0144
	4colors/2×2	0.9984	0.9941	0.9840	0.9821	0.9179	0.9115	0.0163	-0.0018	0.0725
Cd	2colors/1×2	0.9949	0.9843	0.9795	0.9939	0.9959	0.9921	0.0010	-0.0116	-0.0126
	3colors/1×3	0.9961	0.9653	0.9778	0.9836	0.9812	0.9488	0.0125	-0.0159	0.0290
	4colors/2×2	0.9976	0.9845	0.9833	0.9832	0.9914	0.9468	0.0144	-0.0069	0.0364

Table S16. Information entropy of original images and encrypted images $CuInS_2$ and Cd Quantum dot films with different sizes and colors (Grassland scenes, in-situ image encryption).

QD	Film	Original image H（2D）			Encrypted image（2D）			ΔH（2D）		
		R	G	B	R	R	G	B	G	R
$CuInS_2$	2colors/1×2	6.8387	7.7584	6.4411	0.5897	0	7.7653	-6.2490	-7.7584	1.3242
	3colors/1×3	6.9800	7.9195	6.7913	0.1170	0	8.5213	-6.8630	-7.9195	1.7600
	4colors/2×2	6.9275	7.8520	6.6250	3.0524	0	9.1909	-3.8751	-7.8520	2.5659
Cd	2colors/1×2	7.1737	8.1797	7.1839	2.5313	0	7.8753	-4.6424	-8.1797	0.6914
	3colors/1×3	7.1744	8.2504	7.3997	0.0192	0	8.5716	-7.1552	-8.2504	1.1719
	4colors/2×2	7.2885	8.3004	7.341	3.3936	0	8.9669	-3.8949	-8.3004	1.6259

Table S17. The PSNR of in-situ image encryption using $CuInS_2$ QD and Cd QD films

Scene contents	QD	QD film	PSNR (dB)
Gobi scenes	$CuInS_2$	4 colors/2×4	37.16
		4 colors/4×4	38.95
		6colors /4×4	38.09
	Cd	4 colors/2×4	34.90
		4 colors /4×4	33.51
		6 colors /4×4	30.89
Forest scenes	$CuInS_2$	2 colors /1×2	32.60
		3 colors /1×3	34.02
		4 colors /2×2	30.36
	Cd	2colors /1×2	43.84
		3 colors /1×3	46.13
		4 colors/2×2	45.17
Grassland scenes	$CuInS_2$	2 colors/1×2	26.38
		3 colors/1×3	24.18
		4 colors/2×2	26.12
	Cd	2 colors /1×2	39.63
		3 colors /1×3	43.16
		4 colors /2×2	40.03
Woodland scenes	$CuInS_2$	2 colors /1×2	32.21
		3 colors/1×3	35.18
		4 colors/2×2	32.53
	Cd	2 colors /1×2	48.04
		3 colors/1×3	51.54
		4 colors /2×2	42.83

Table S18. Information entropy of original images and key images using $CuInS_2$ QD film and Cd QD film (Desert scene; in-situ image encryption).

QD	QD film	Image	Gray image H(2D)			Color image H(2D)
			R	G	B	
$CuInS_2$	4 colors /2×4	Original image 1	7.4015	8.0455	7.2109	22.6579
		Key image 1	0	0.0275	7.6289	7.6564
	4 colors /4×4	Original image 2	7.2073	7.8450	7.0290	22.0813
		Key image 2	0	0.0475	7.6879	7.7354
	6 colors /4×4	Original image 3	7.0900	7.7008	6.8931	21.6839
		Key image 3	0	0.9070	8.0143	8.9213
Cd	4 colors /2×4	Original image 4	7.3551	7.7901	7.2051	22.3503
		Key image 4	0	4.8427	8.1966	13.0393
	4 colors /4×4	Original image 5	7.3470	7.8874	7.2351	22.4695
		Key image 5	0	0.2857	7.6420	7.9277
	6 colors /4×4	Original image 6	7.0343	7.4484	7.1261	21.6088
		Key image 6	0	1.5229	8.0499	9.5728

Table S19. Information entropy of original images and key images using $CuInS_2$ QD film and Cd QD film (Gobi scenes; in-situ image encryption).

QD	QD film	Image	Gray image H(2D)			Color image H(2D)
			R	G	B	
$CuInS_2$	4 colors /2×4	Original image 7	6.0733	6.5765	5.9971	18.6469
		Key image 7	0	0.6841	7.5613	8.2454
	4 colors /4×4	Original image 8	6.1581	6.6876	6.0362	18.8819
		Key image 8	0	0.0911	7.3794	7.4705
	6 colors /4×4	Original image 9	5.9540	6.4943	5.6814	18.1297
		Key image 9	0	0.1355	7.4379	7.5734
Cd	4 colors /2×4	Original image 10	5.8551	6.1740	5.6961	17.7252
		Key image 10	0	4.3022	6.8499	11.1521
	4 colors /4×4	Original image 11	5.8015	6.1482	6.0449	17.9946
		Key image 11	0	0.0197	7.5369	7.5566
	6 colors /4×4	Original image 12	6.0381	6.4129	5.9093	18.3603
		Key image 12	0	0.0239	7.7646	7.7885

Table S20. Information entropy of original images and key images using $CuInS_2$ QD film and Cd QD film (Forest scenes; in-situ image encryption)

QD	QD film	Image	Gray image H(2D)			Color image H(2D)
			R	G	B	
$CuInS_2$	2 colors /1×2	Original image 13	6.0733	6.5765	5.9971	18.6469
		Key image 13	0	0.6841	7.5613	8.2454
	3 colors /1×3	Original image 14	6.1581	6.6876	6.0362	18.8819
		Key image 14	0	0.0911	7.3794	7.4705
	4 colors /2×2	Original image 15	5.9540	6.4943	5.6814	18.1297
		Key image 15	0	0.1355	7.4379	7.5734
Cd	2 colors /1×2	Original image 16	5.8551	6.1740	5.6961	17.7252
		Key image 16	0	4.3022	6.8499	11.1521
	3 colors /1×3	Original image 17	5.8015	6.1482	6.0449	17.9946
		Key image 17	0	0.0197	7.5369	7.5566
	4 colors /2×2	Original image 18	6.0381	6.4129	5.9093	18.3603
		Key image 18	0	0.0239	7.7646	7.7885

Table S21. Information entropy of original images and key images using $CuInS_2$ QD film and Cd QD film (Grassland scenes; in-situ image encryption)

QD	QD film	Image	Gray image H(2D)			Color image H(2D)
			R	G	B	
$CuInS_2$	2 colors /1×2	Original image 19	5.8890	6.0070	5.6380	17.5340
		Key image 19	5.4194	0.0308	9.2233	14.6735
	3 colors /1×3	Original image 20	5.9946	6.7181	5.7760	18.1587
		Key image 20	0.0393	0	7.9138	7.9777
	4 colors /2×2	Original image 21	5.7477	6.4299	5.5131	17.6877
		Key image 21	4.5444	0	7.7023	12.2467
Cd	2 colors /1×2	Original image 22	6.8399	7.7849	6.7350	21.3598
		Key image 22	3.5552	0	8.5198	12.0750
	3 colors /1×3	Original image 23	6.7496	7.6099	6.5058	20.8653
		Key image 23	1.2444	0	8.9402	10.1846
	4 colors /2×2	Original image 24	6.6271	7.6112	6.5660	20.8043
		Key image 24	2.4045	0	8.2055	10.6100

Table S22. Information entropy of original images and key images using $CuInS_2$ QD film and Cd QD film (Woodland scenes; in-situ image encryption)

QD	QD film	Image	Gray image H(2D)			Color image H(2D)
			R	G	B	
$CuInS_2$	2 colors /1×2	Original image 25	6.8387	7.7584	6.4411	21.0382
		Key image 25	4.2214	0	9.2630	13.4844
	3 colors /1×3	Original image 26	6.9800	7.9195	6.7913	21.6908
		Key image 26	3.7428	0	8.6073	12.3501
	4 colors /2×2	Original image 27	6.9275	7.8520	6.6250	21.4045
		Key image 27	1.7466	0	8.0134	9.7600
Cd	2 colors /1×2	Original image 28	7.1737	8.1797	7.1839	22.5373
		Key image 28	3.4634	0	9.1026	12.5660
	3 colors /1×3	Original image 29	7.1744	8.2504	7.3997	22.8245
		Key image 29	0.0195	0	8.591	8.6105
	4 colors /2×2	Original image 30	7.2885	8.3004	7.341	22.9299
		Key image 30	2.6490	0	8.1646	10.7954

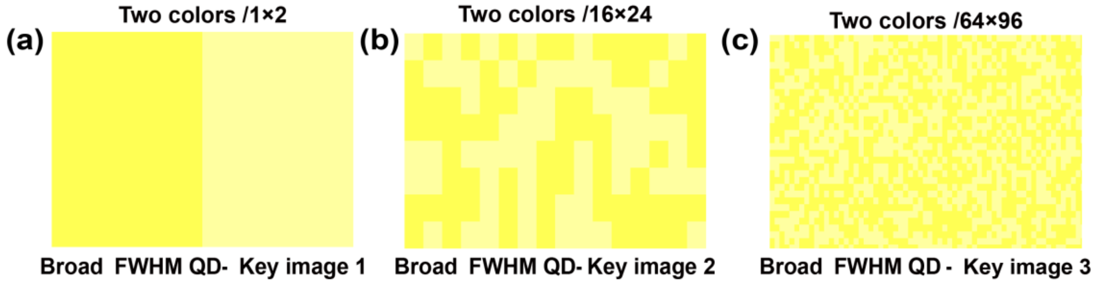

Figure S1. The key images of simulating in situ image encryption using QD films of different sizes. (a-c) The key images of simulating in-situ image encryption using QD films of Broad FWHM/two colors/1×2, Broad FWHM/Two colors/16×24, and Broad FWHM/Two colors/64×96, respectively.

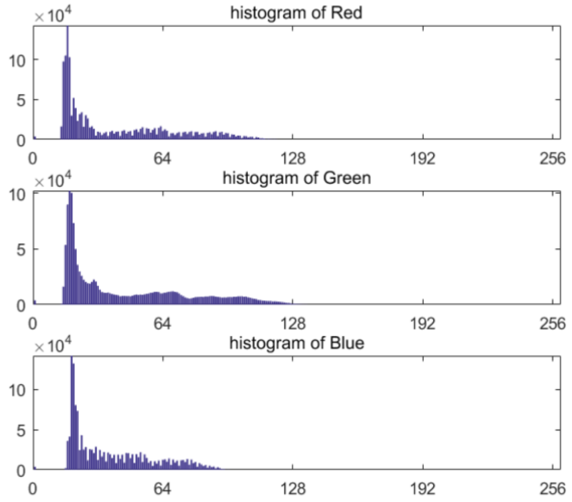

Figure S2. Histogram of original images for simulating in situ image encryption

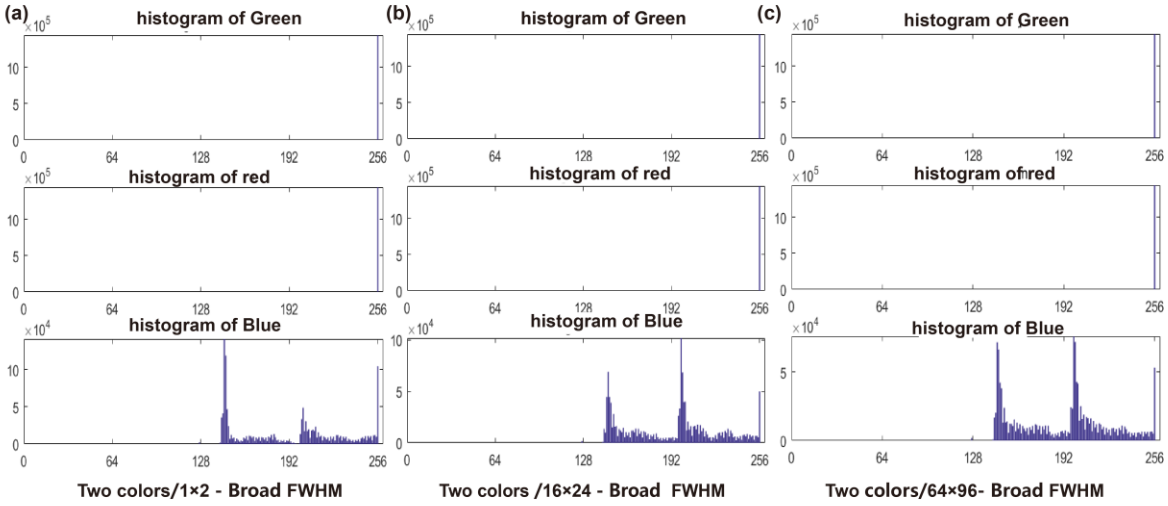

Figure S3. Histogram of encrypted images using QD film of different sizes (simulating in situ image encryption). (a)-(c) Histogram of simulated encrypted images using QD film of Broad FWHM/Two colors/1×2, Broad FWHM/Two colors/16×24, and Broad FWHM/Two colors/64×96, respectively.

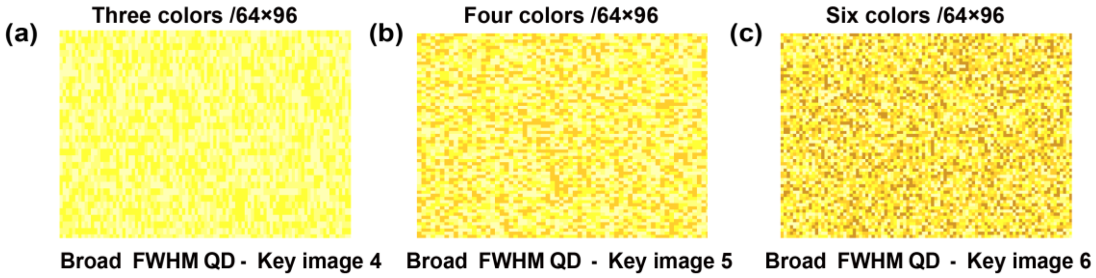

Figure S4. The key images using different QD film of color (simulating in situ image encryption). (a-c) A series of simulated key images using QD film of Broad FWHM/Three colors/64×96, Broad FWHM/Four colors/16×24, and Broad FWHM/Six colors/64×96, respectively.

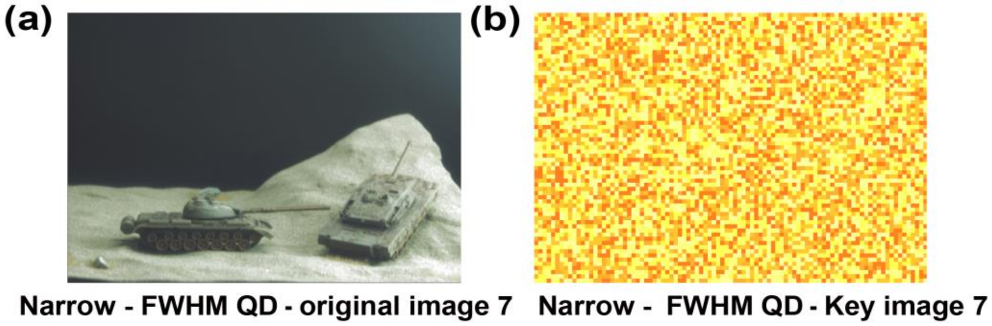

Figure S5. The Original image and key image using QD film of narrow FWHM/Six colors/64×96) (simulating in situ image encryption). (a) The Original image using QD film of narrow FWHM/Six colors/64×96) (b) The key image using QD film of narrow FWHM/Six colors/64×96) (simulating in-situ image encryption)

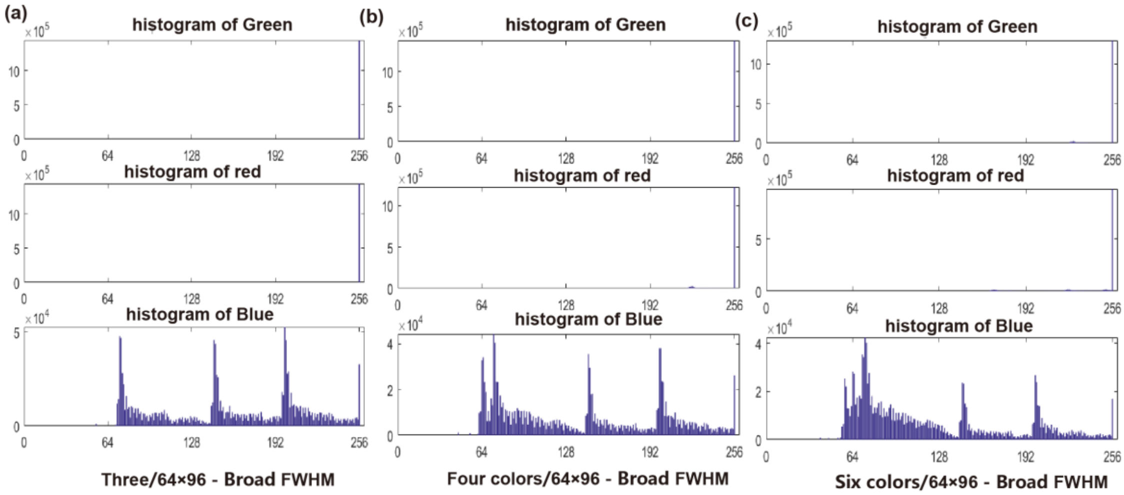

Figure S6. Histogram of encrypted images using QD film of different colors (simulating in situ image encryption) (a)-(c) Histogram of simulated encrypted images with Broad FWHM/Three colors/64×96, Broad FWHM/Four colors/64×96, and Broad FWHM/Six colors/64×96, respectively.

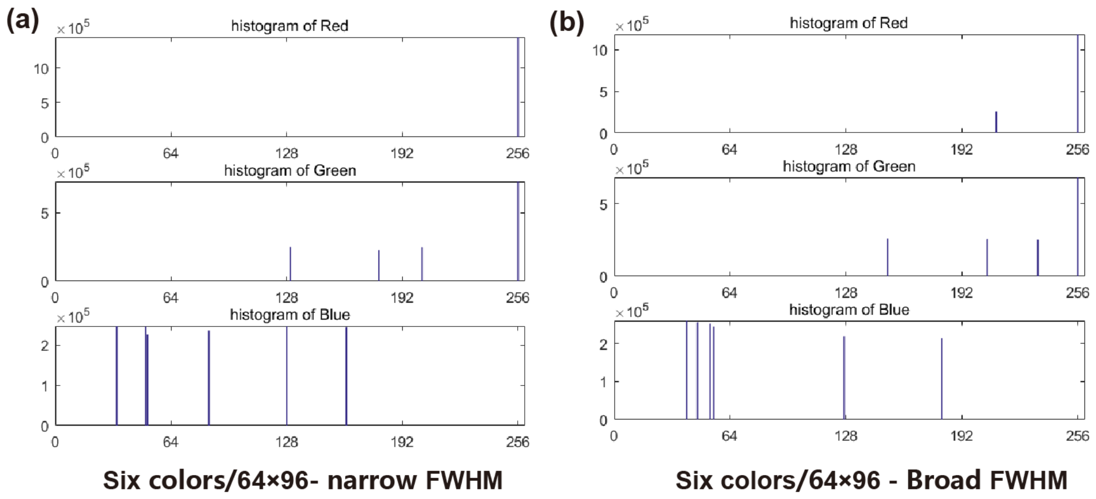

Figure S7. Histogram of key images using QD film of different FWHM.(simulating in situ image encryption) (a)-(b) Histogram of simulated key images with narrow FWHM/six colors/64×96, and Broad FWHM/six colors/64×96, respectively.

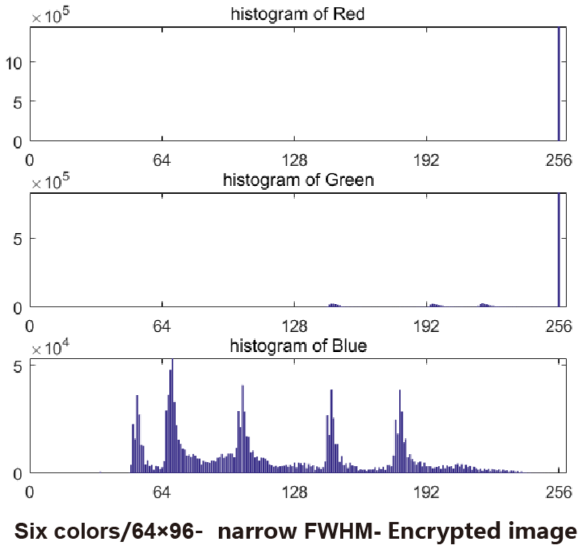

Figure S8. Histogram of encrypted image using narrow FWHM/six colors/64×96 (simulating in situ image encryption).

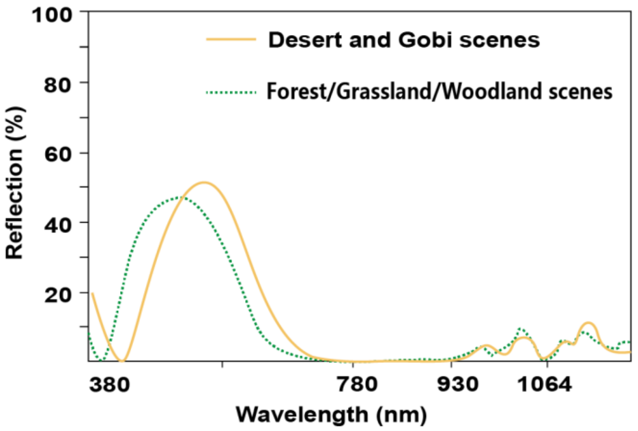

Figure S9. Reflectance spectra of desert scenes, Gobi scenes, forest scenes, grassland scenes, and woodland scenes.

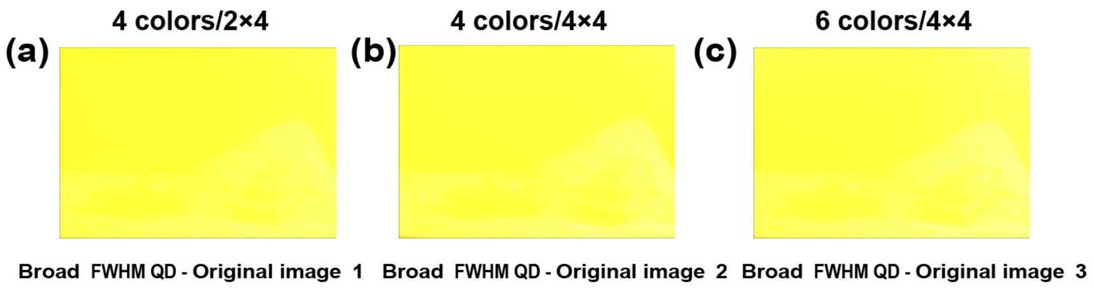

Figure S10. The key images of in-situ image encryption using $CuInS_2$ QD film (a) The key image of in situ image using Broad FWHM/4 colors/2×4 QD film. (b) The key image of in-situ image using Broad FWHM/4 colors/4×4 QD film. (c) The key image of in-situ image using Broad FWHM/6 colors/4×4 QD film.

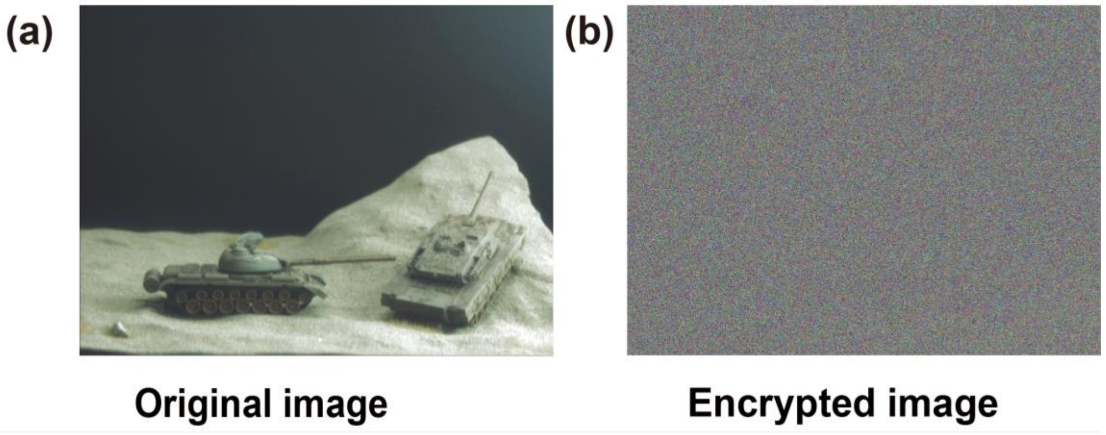

Figure S11. The encrypted simulation results. (a) The original image using high resolution patterned QD film. (b) The encrypted simulation results using high resolution patterned QD film.

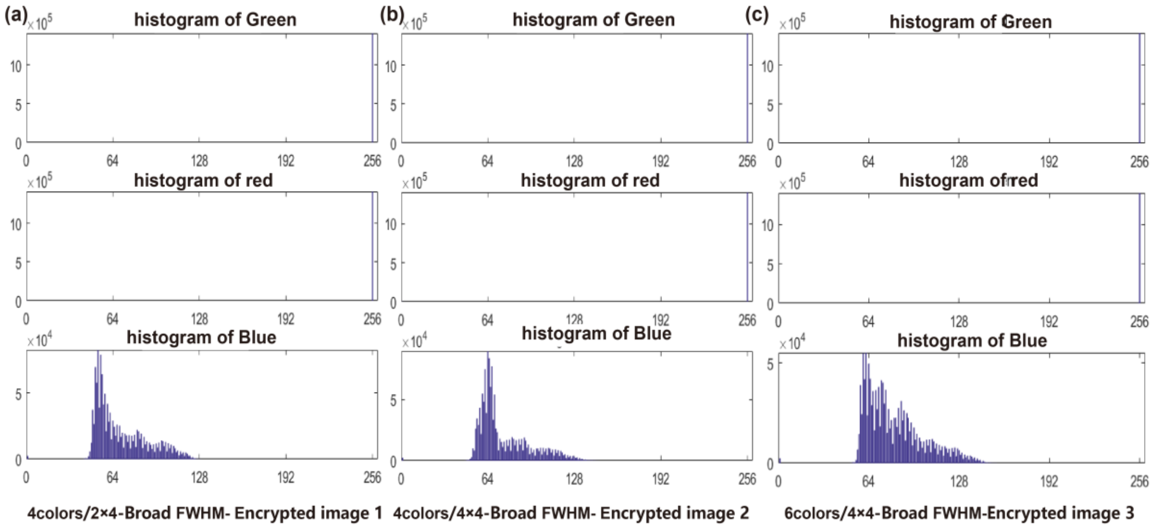

Figure S12. Histogram of encrypted images using $CuInS_2$ QD film (in situ image encryption). (a) Histogram of encrypted image using Broad FWHM/4 colors/2×4 QD film. (b) Histogram of encrypted image using Broad FWHM/4 colors/4×4 QD. (c) Histogram of encrypted image using Broad FWHM/6 colors/4×4 QD.

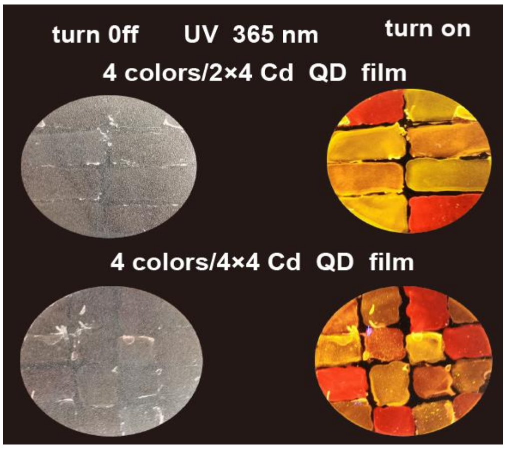

Figure S13. The photographs of 4 colors /2×4 Cd QD film, and 4 colors /4×4 Cd QD film under daylight and 365 nm UV lamp using Cd QD material.

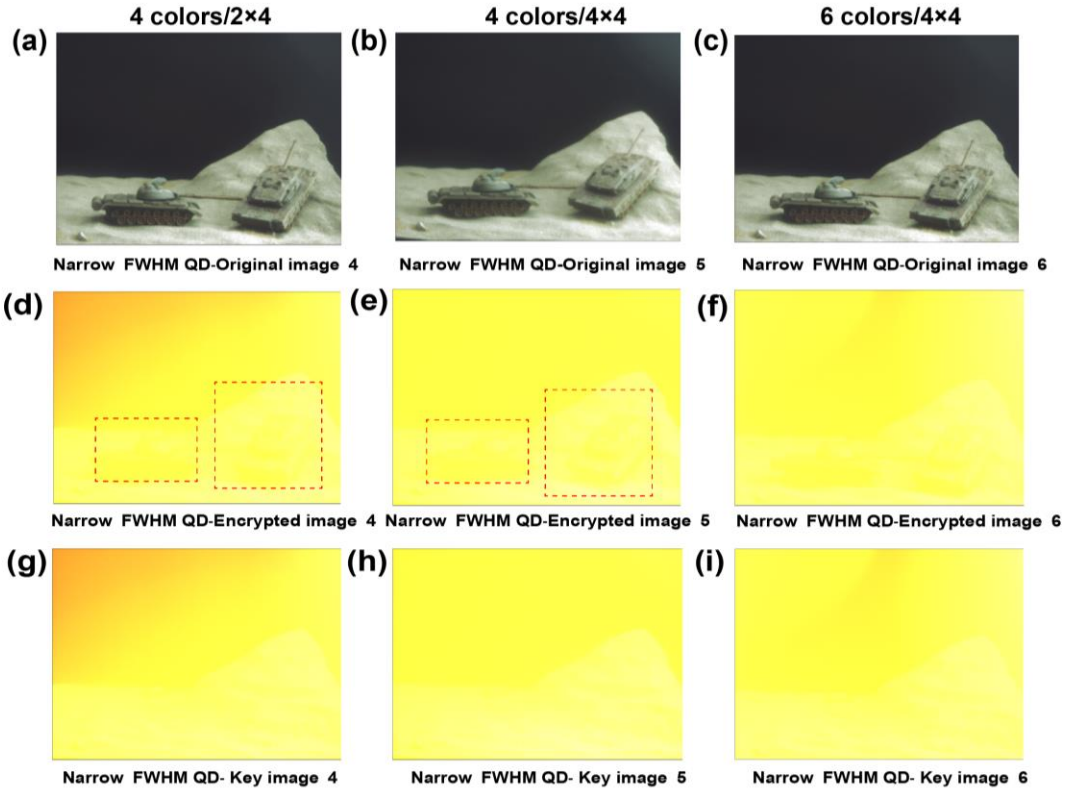

Figure S14. The encryption results of in situ image encryption using Cd QD film. (a-c) Original image of in-situ image encryption using Narrow FWHM/4 colors/2×4 QD film, Narrow FWHM/4 colors/4×4 QD film and Narrow FWHM/6 colors/4×4 QD film. (d-f) Encrypted image of in-situ image encryption using Narrow FWHM/4 colors/2×4 QD film, Narrow FWHM/4 colors/4×4 QD film and Narrow FWHM/6 colors/4×4 QD film. (g-i) Key image of in-situ image encryption using Narrow FWHM/4 colors/2×4 QD film, Narrow FWHM/4 colors/4×4 QD film and Narrow FWHM/6 colors/4×4 QD film.

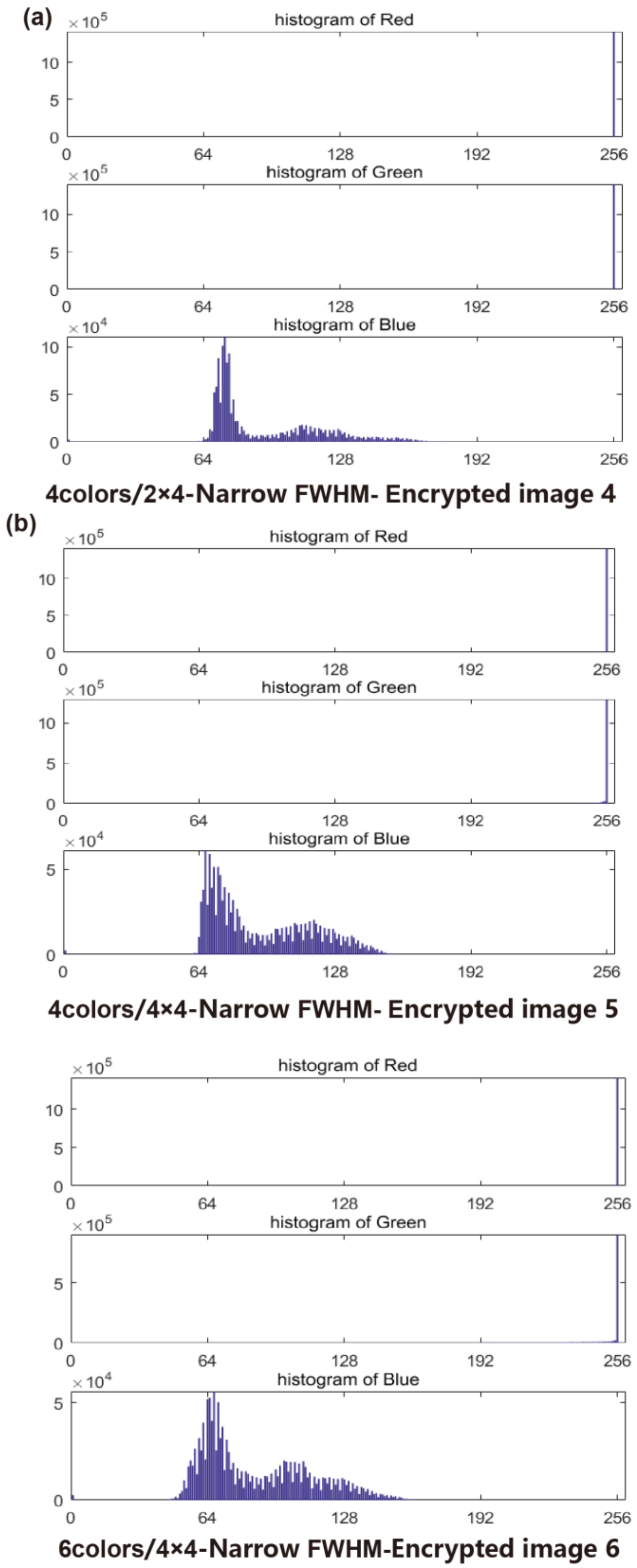

Figure S15. Histogram of encrypted images of in situ image encryption using Cd QD film. (a) Histogram of encrypted image using narrow FWHM/4 colors/2×4 QD film. (b) Histogram of encrypted image using narrow FWHM/4 colors/4×4 QD film. (c) Histogram of encrypted image using narrow FWHM/6 colors/4×4 QD film.

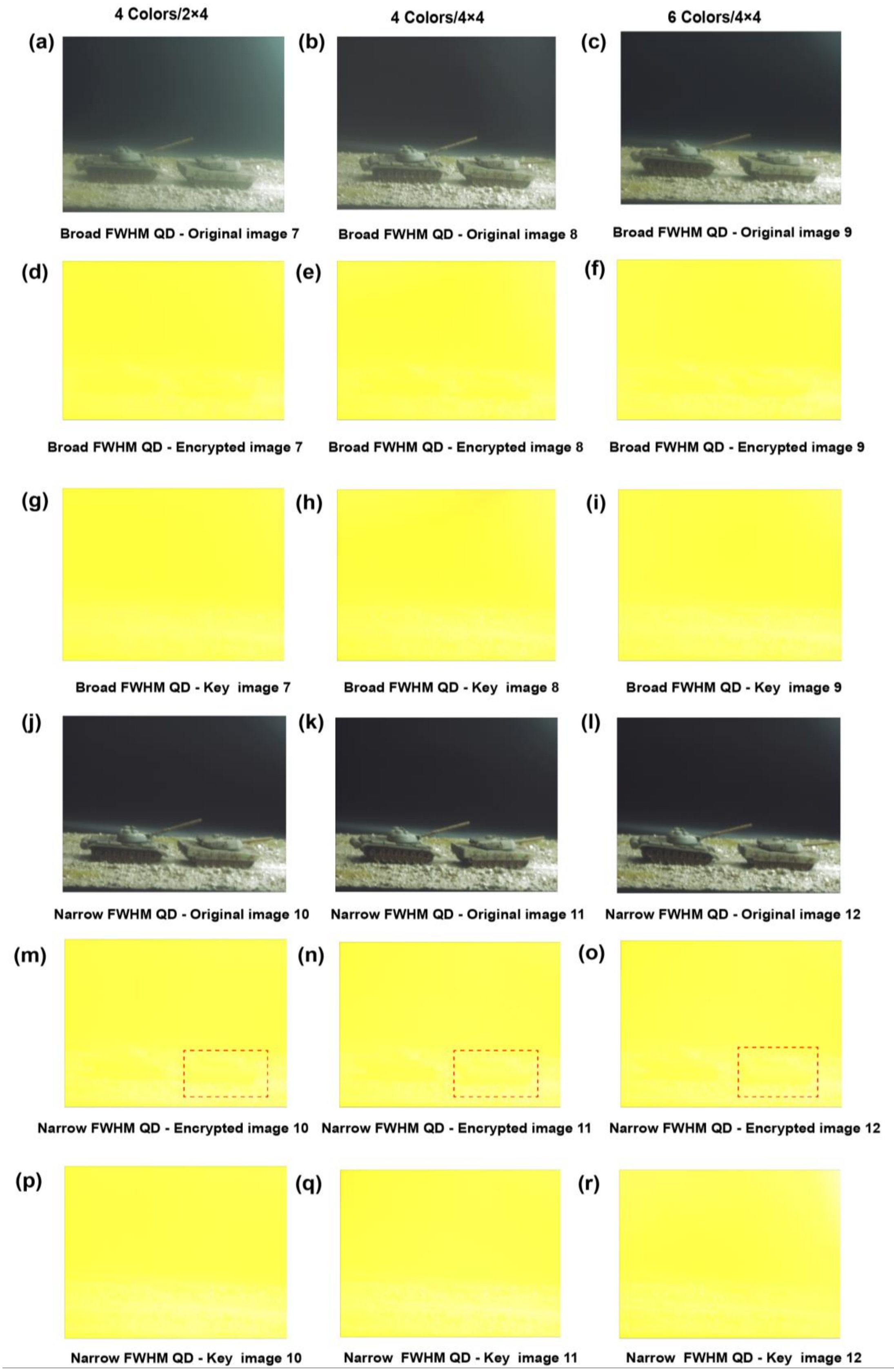

Figure S16. The encryption results using $CuInS_2$ QD film and Cd QD film (Gobi scenes; in situ natural image encryption) (a-i) Original image, encrypted image and key image of i using $CuInS_2$ QD film (j-r) Original image, encrypted image and Key image using Cd QD film

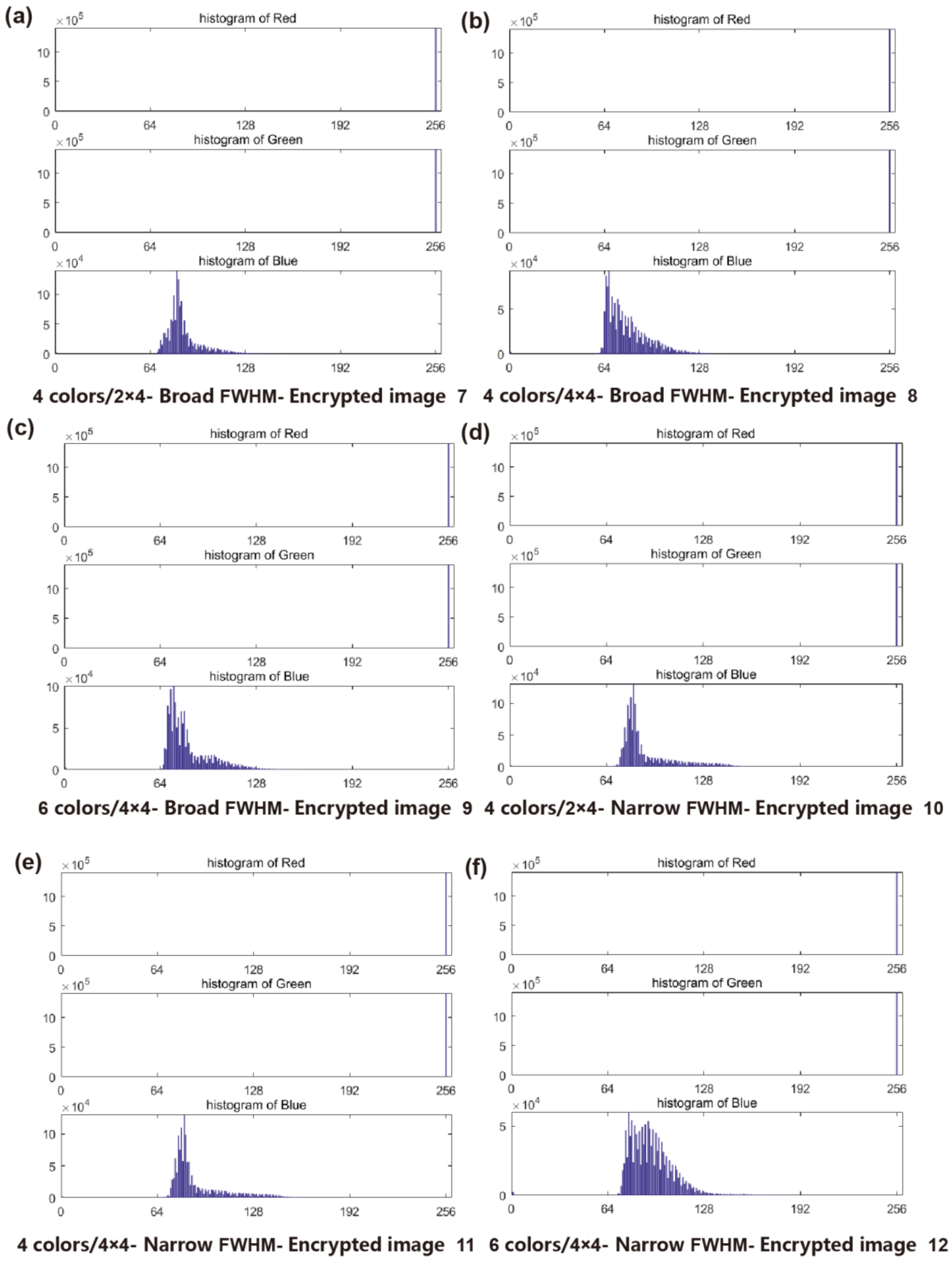

Figure S17. Histogram of encrypted images using $CuInS_2$ and Cd QD film (Gobi scenes; in situ image encryption). (a-c) Histogram of encrypted image using $CuInS_2$ QD film (4 colors/2×4 QD film, 4 colors/4×4 QD film and 6 colors/4×4 QD film). (d-f) Histogram of encrypted image using Cd QD film (4 colors/2×4 QD film, 4 colors/4×4 QD film and 6 colors/4×4 QD film).

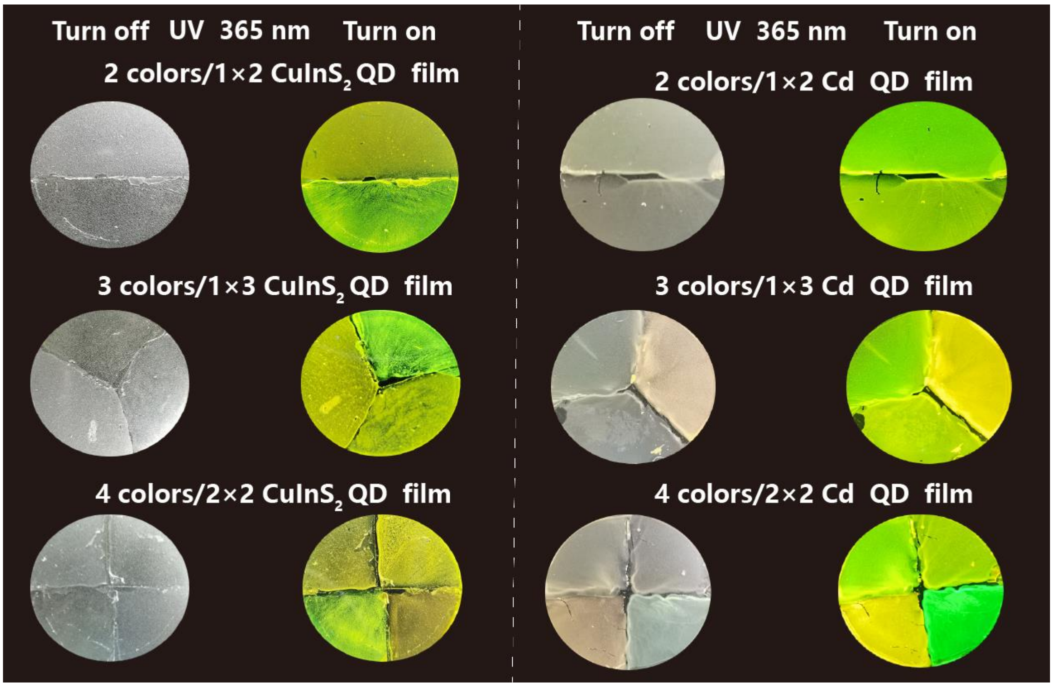

Figure S18. The photographs of 2 colors /1×2 $CuInS_2$ QD film, 3 colors /1×3 $CuInS_2$ QD film and 4 colors/2×2 $CuInS_2$ QD film under daylight and 365 nm UV lamp using Cd QD material.

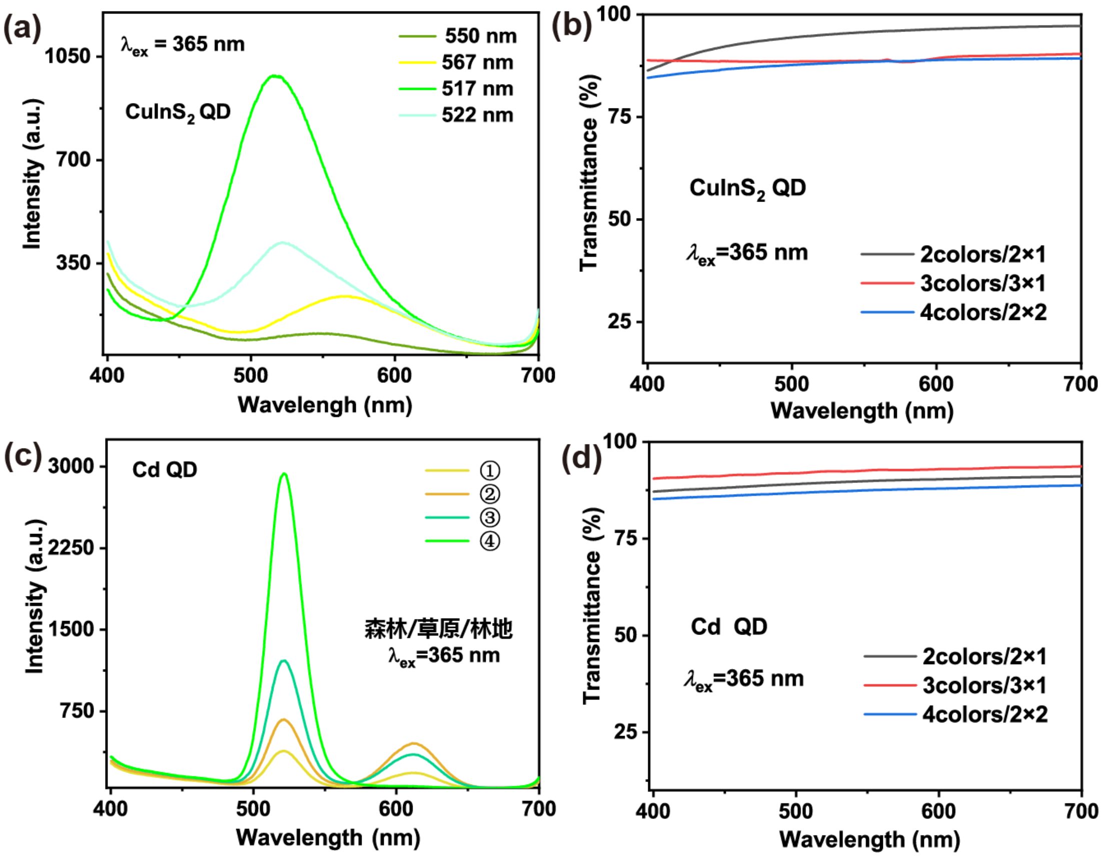

Figure S19. Photoluminescence emission spectra and transmittance spectra of different QD films. (a-b) Photoluminescence emission spectra and transmittance spectra of different $CuInS_2$ QD film (c-d) Photoluminescence emission spectra transmittance spectra of different Cd QD film.

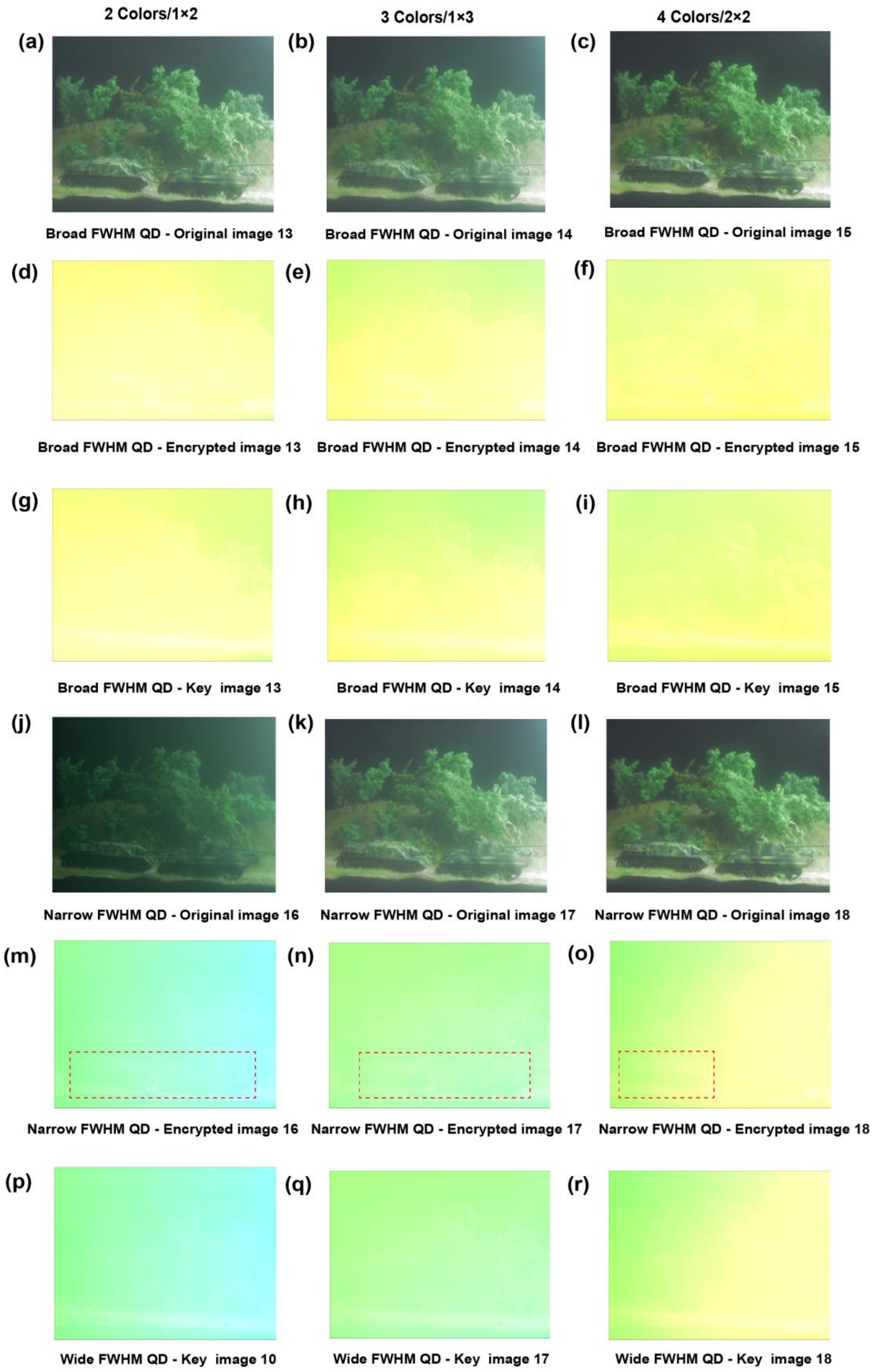

Figure S20. The encryption results of using $CuInS_2$ QD film and Cd QD film (Forest scenes; in situ image encryption). (a-i) Original image, encrypted image and key image of using $CuInS_2$ QD film. (j-r) Original image, encrypted image and Key image using Cd QD film

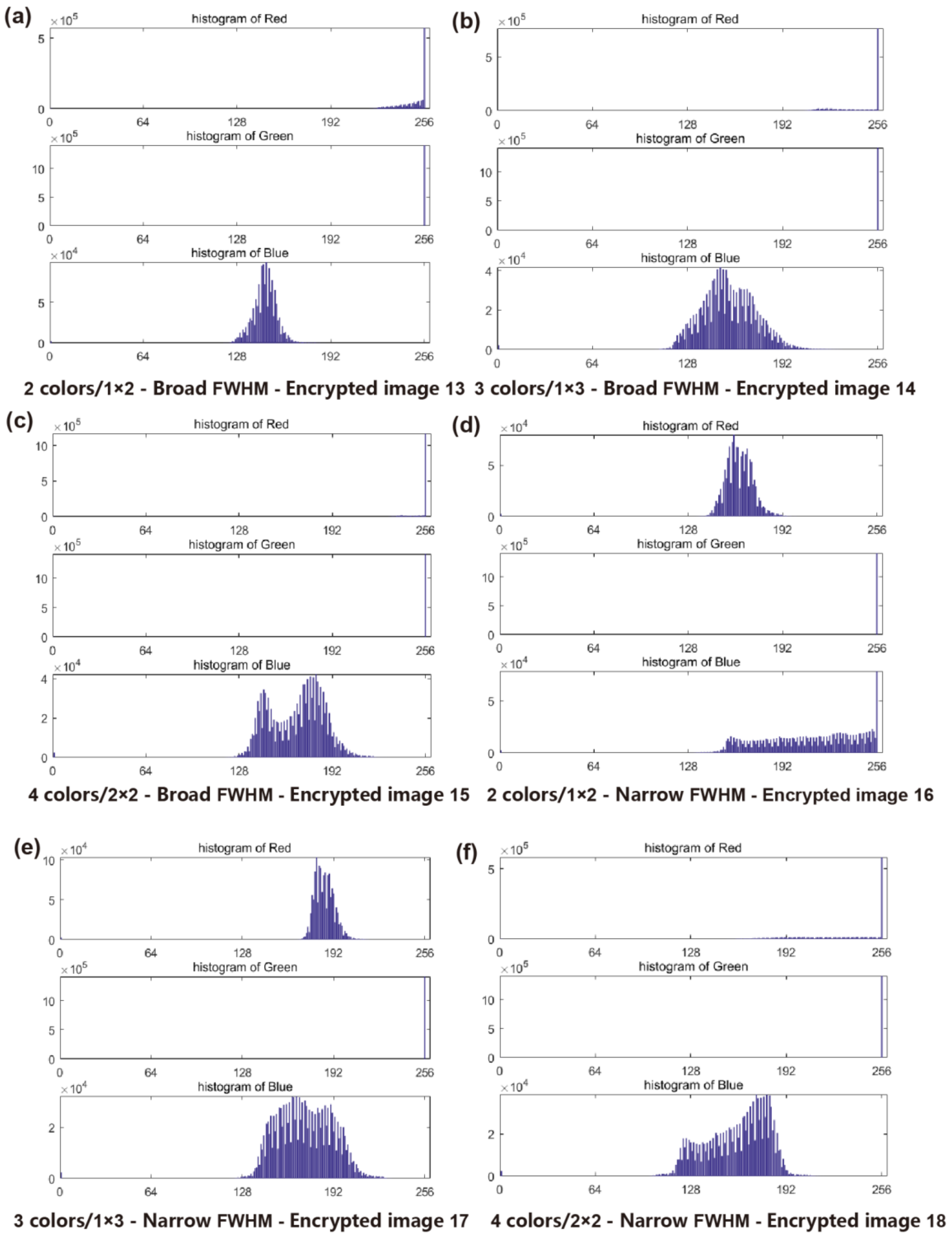

Figure S21. Histogram of encrypted images using $CuInS_2$ and Cd QD film (Forest scenes; in situ natural image encryption). (a-c) Histogram of encrypted image using $CuInS_2$ QD film (4 colors/2×4 QD film, 4 colors/4×4 QD film and 6 colors/4×4 QD film). (d-f) Histogram of encrypted image using Cd QD film (4 colors/2×4 QD film, 4 colors/4×4 QD film and 6 colors/4×4 QD film).

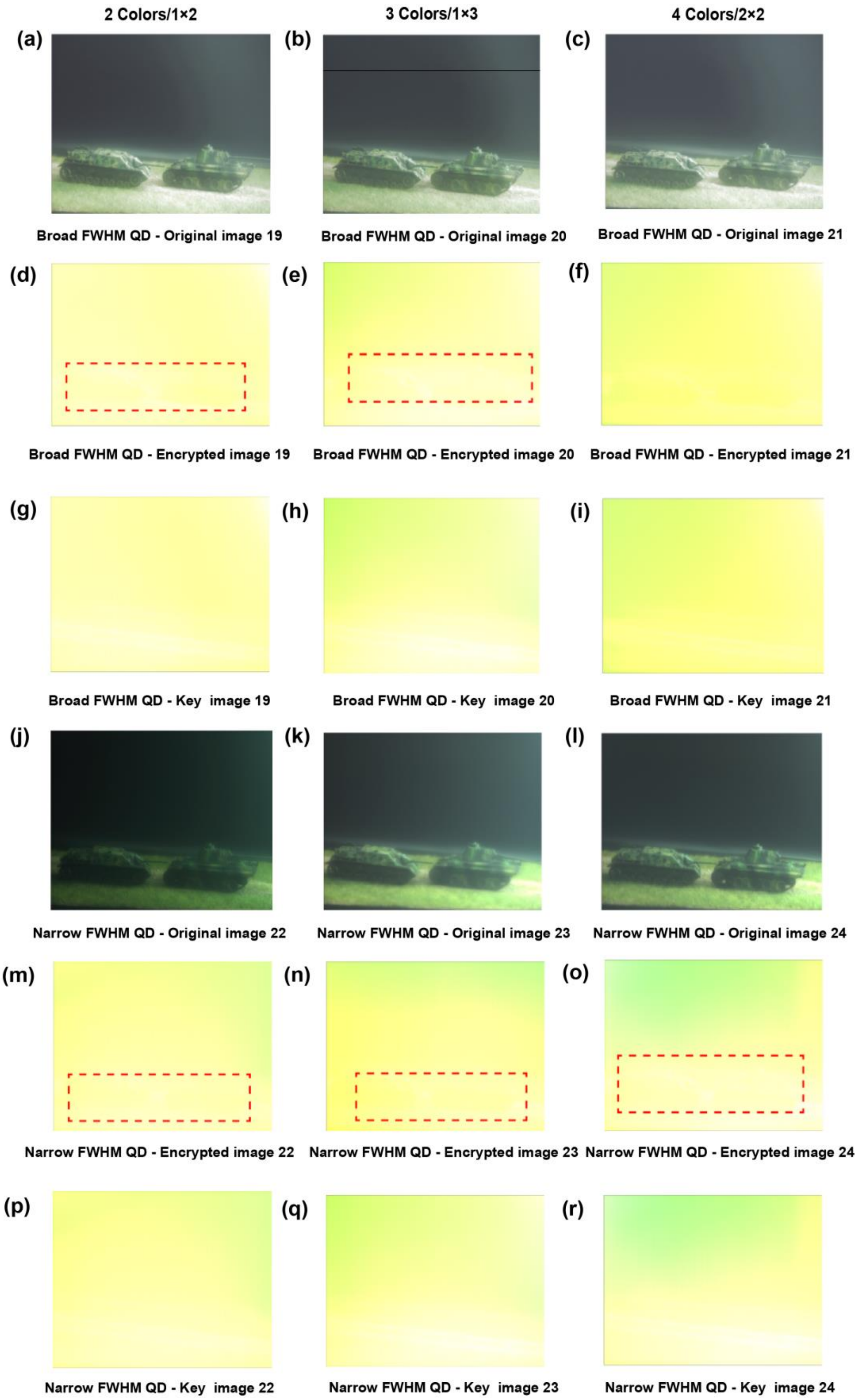

Figure S22. The encryption results using $CuInS_2$ QD film and Cd QD film (Grassland scenes; in situ image encryption). (a-i) Original image, encrypted image and key image using $CuInS_2$ QD film. (j-r) Original image, encrypted image and Key image using Cd QD film.

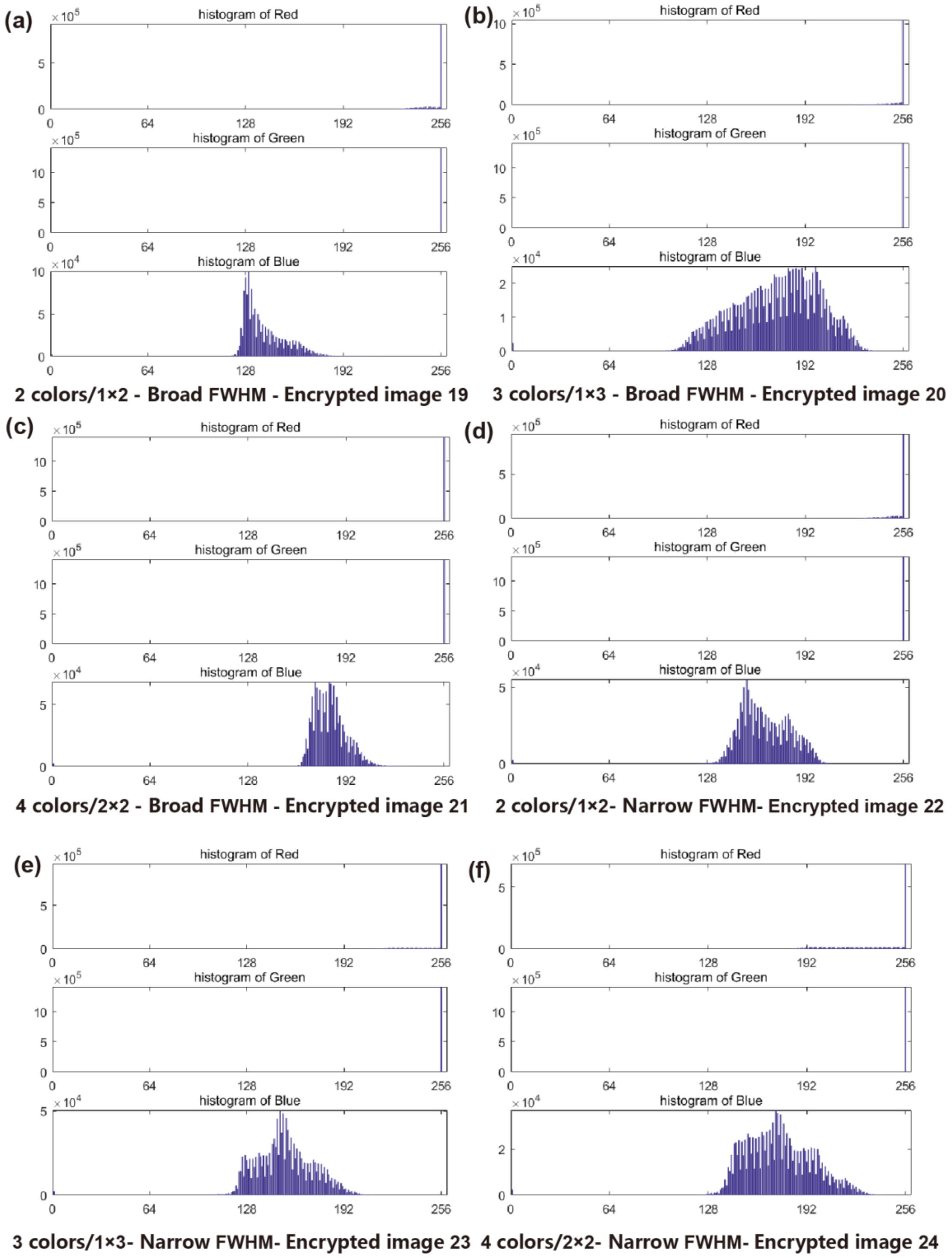

Figure S23. Histogram of encrypted images encryption using $CuInS_2$ and Cd QD film (Grassland scenes; in situ natural image encryption). (a-c) Histogram of encrypted image using $CuInS_2$ QD film (4 colors/2×4 QD film, 4 colors/4×4 QD film and 6 colors/4×4 QD film). (d-f) Histogram of encrypted image using Cd QD film (4 colors/2×4 QD film, 4 colors/4×4 QD film and 6 colors/4×4 QD film).

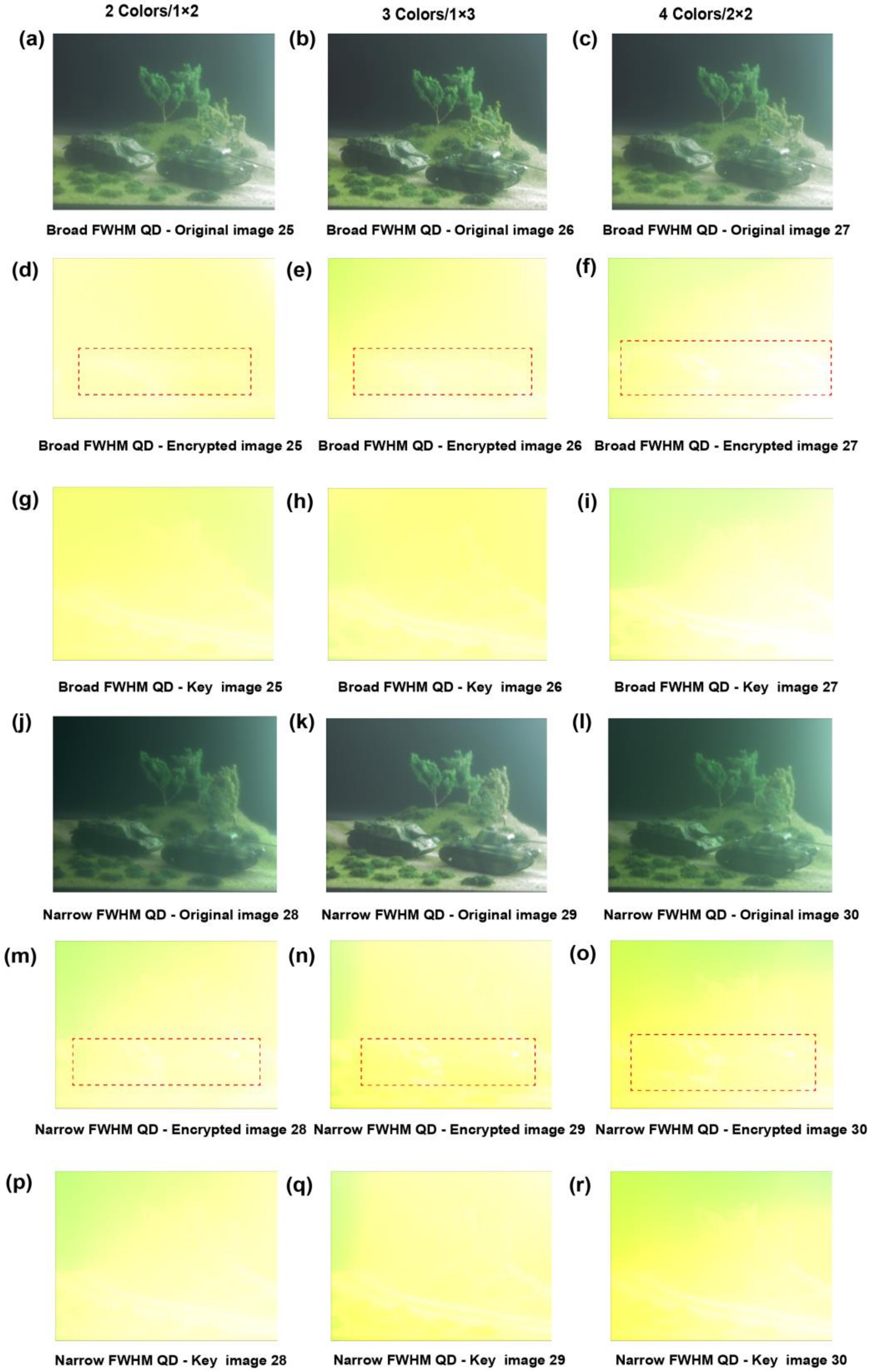

Figure S24. The encryption results using $CuInS_2$ QD film and Cd QD film (Woodland scene; in situ image encryption). (a-i) Original image, encrypted image and key image using $CuInS_2$ QD film. (j-r) Original image, encrypted image and Key image of in-situ image encryption using Cd QD film.

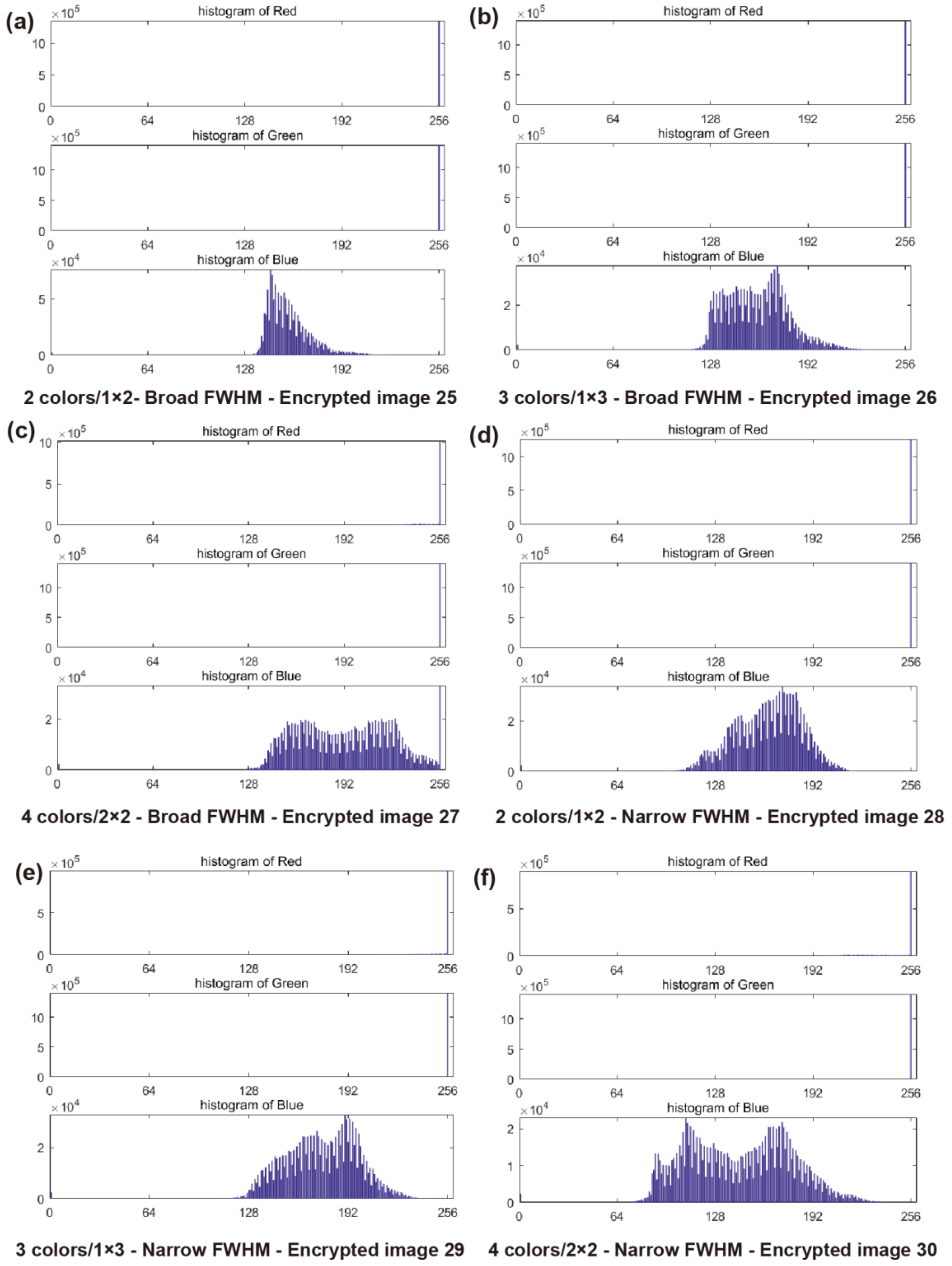

Figure S25. Histogram of encrypted images using $CuInS_2$ and Cd QD film (Woodland scene; in situ natural image encryption). (a-c) Histogram of encrypted image using $CuInS_2$ QD film (4 colors/2×4 QD film, 4 colors/4×4 QD film and 6 colors/4×4 QD film). (d-f) Histogram of encrypted image using Cd QD film (4 colors/2×4 QD film, 4 colors/4×4 QD film and 6 colors/4×4 QD film).

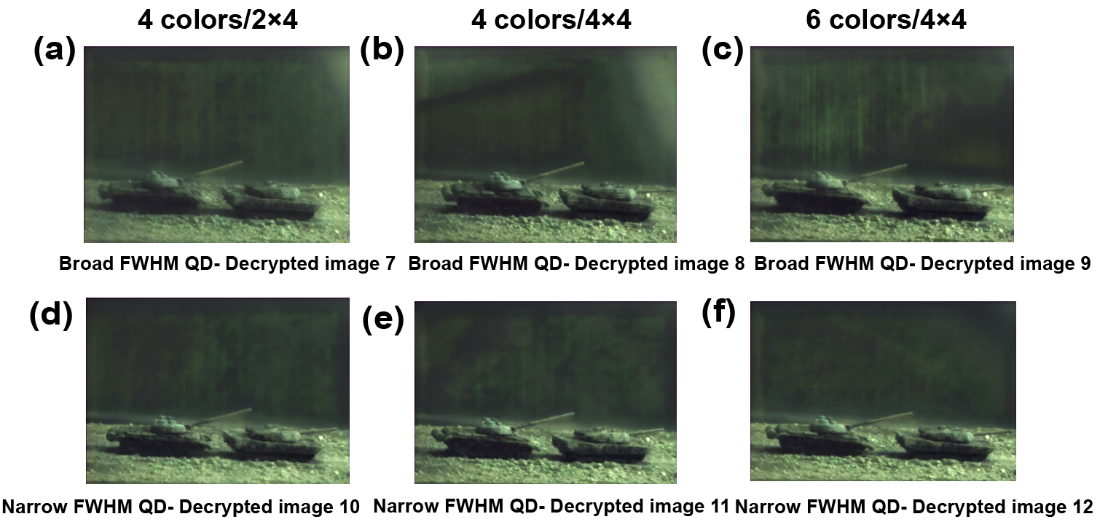

Figure S26. The decryption results using $CuInS_2$ QD and Cd QD films (Gobi scenes; in situ image encryption). (a-c) The decryption results using $CuInS_2$ QD (4 colors/2×4 QD film, 4 colors/4×4 QD film and 6 colors/4×4 QD film). (d-f) The decryption results using Cd QD (4 colors/2×4 QD film, 4 colors/4×4 QD film and 6 colors/4×4 QD film).

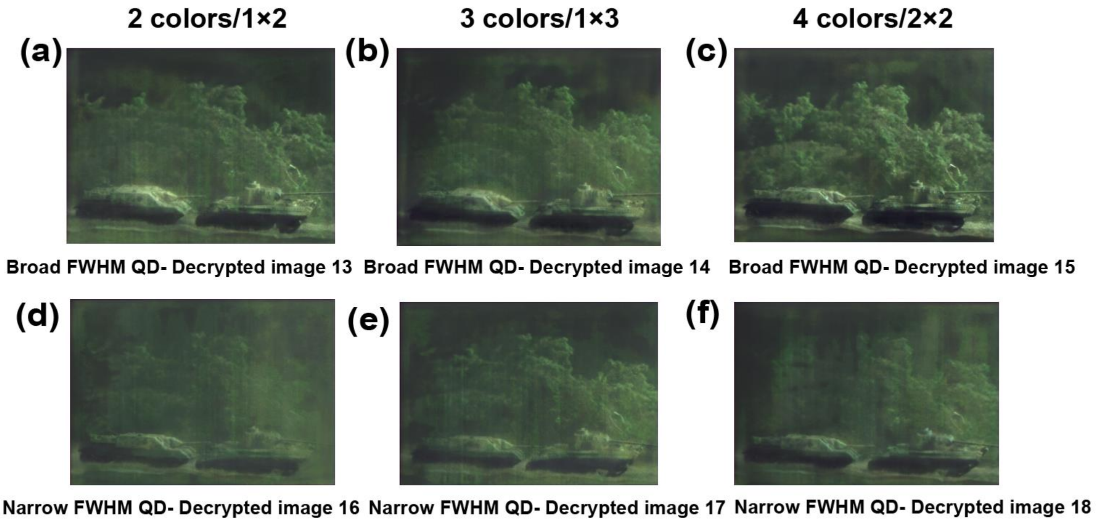

Figure S27. The decryption results using $CuInS_2$ QD and QD Cd films (Forest scenes; in situ image encryption). (a-c) The decryption results using $CuInS_2$ QD (4 colors/2×4 QD film, 4 colors/4×4 QD film and 6 colors/4×4 QD film). (d-f) The decryption results using Cd QD (4 colors/2×4 QD film, 4 colors/4×4 QD film and 6 colors/4×4 QD film).

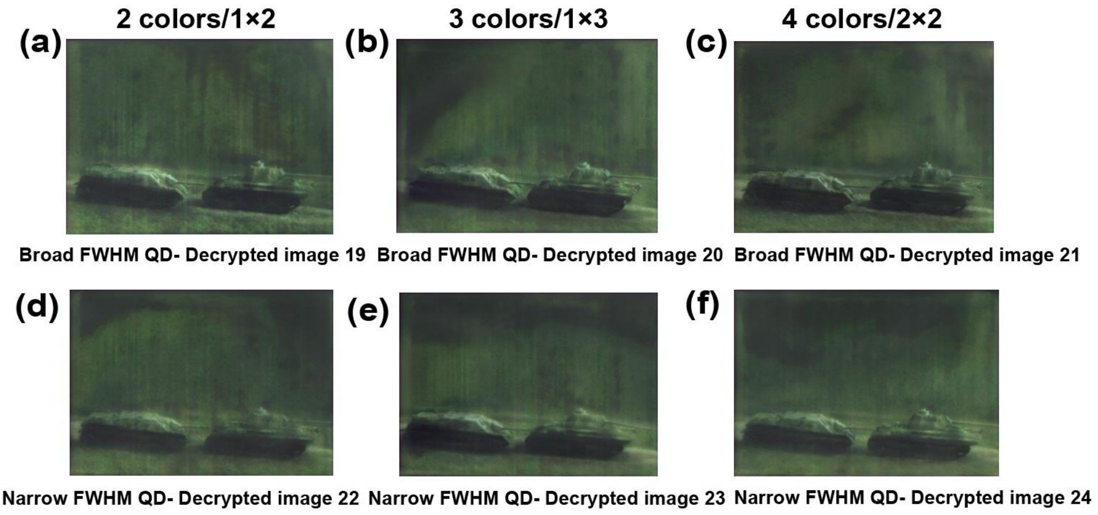

Figure S28. The decryption results using $CuInS_2$ QD and Cd QD film (Grassland scenes; in situ image encryption). (a-c) The decryption results using $CuInS_2$ QD (4 colors/2×4 QD film, 4 colors/4×4 QD film and 6 colors/4×4 QD film). (d-f) The decryption results using Cd QD (4 colors/2×4 QD film, 4 colors/4×4 QD film and 6 colors/4×4 QD film).

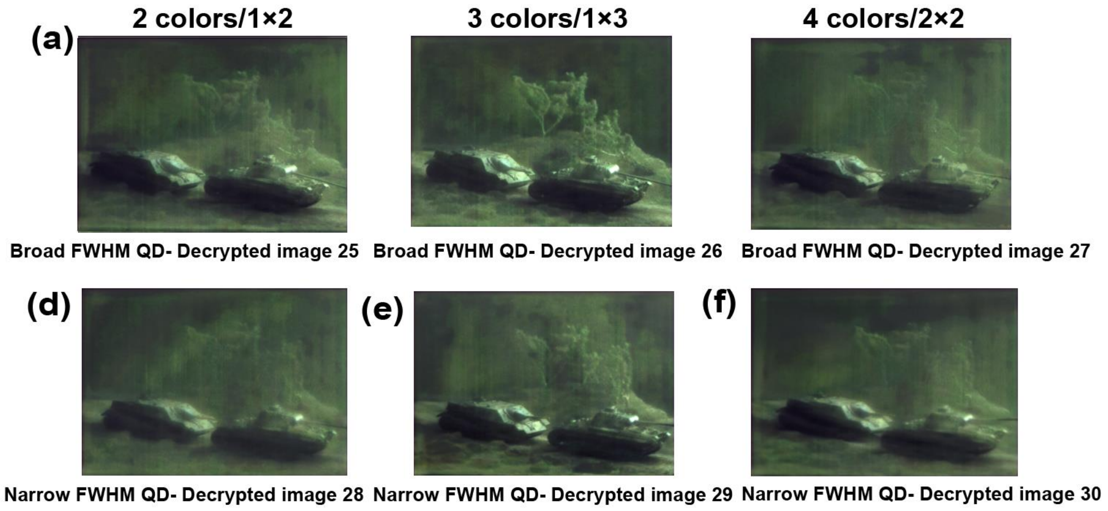

Figure S29. The decryption results of using $CuInS_2$ QD and Cd QD film (Woodland scenes; in situ image encryption). (a-c) The decryption results using $CuInS_2$ QD (4 colors/2×4 QD film, 4 colors/4×4 QD film and 6 colors/4×4 QD film). (d-f) The decryption results using Cd QD (4 colors/2×4 QD film, 4 colors/4×4 QD film and 6 colors/4×4 QD film).
